# Antibiotic Prophylaxis Strategies and Surgical Site Infections in Colorectal Surgery: A Systematic Review and Network Meta-Analysis

**DOI:** 10.1001/jamanetworkopen.2025.60095

**Published:** 2026-02-19

**Authors:** Shahrzad Motaghi, Samer G. Karam, Francesca Mulazzani, Fatemeh Mirzayeh Fashami, Tayler A. Buchan, Sara Ibrahim, Shahryar Moradi Falah Langeroodi, Sahar Khademioore, Rachel J. Couban, Lawrence Mbuagbaw, Dominik Mertz, Mark Loeb

**Affiliations:** 1 Department of Health Research Methods, Evidence, and Impact, McMaster University, Hamilton, Ontario, Canada; 2 Michael G. DeGroote Cochrane Canada and McMaster GRADE Centre, McMaster University, Hamilton, Ontario, Canada; 3 Department of Medicine and Surgery, University of Milan-Bicocca, Milan, Italy; 4 Anesthesia and Intensive Care Unit, ASST Niguarda Hospital, Milan, Italy; 5 Department of Pharmaceutical Sciences, University of Maryland Eastern Shore, Princess Anne; 6 The Michael G. DeGroote National Pain Centre, McMaster University, Hamilton, Ontario, Canada; 7 Department of Anesthesia, McMaster University, Hamilton, Ontario, Canada; 8 Department of Pediatrics, McMaster University, Hamilton, Ontario, Canada; 9 Research Methodology Centre, The Research Institute of St Joe’s, St Joseph’s Healthcare, Hamilton, Ontario, Canada; 10 Centre for Development of Best Practices in Health, Yaoundé Central Hospital, Yaoundé, Cameroon; 11 Division of Epidemiology and Biostatistics, Department of Global Health, Stellenbosch University, Cape Town, South Africa; 12 Mary Heersink School of Global Health & Social Medicine, McMaster University, Hamilton, Ontario, Canada; 13 Department of Medicine, Division of Infectious Diseases, McMaster University, Hamilton, Ontario, Canada; 14 Department of Pathology and Molecular Medicine, McMaster University, Hamilton, Ontario, Canada

## Abstract

**Importance:**

The optimal choice of antibiotic prophylaxis in elective colorectal surgery remains uncertain, with most reviews emphasizing timing and route of administration rather than direct comparisons of antibiotic classes.

**Objective:**

To compare the outcomes associated with different antibiotic classes and class combinations administered within 24 hours before elective colorectal surgery.

**Data Sources:**

MEDLINE, Embase, Cochrane Central, and Scopus were searched from inception to July 17, 2025.

**Study Selection:**

Eligible studies were randomized clinical trials that enrolled adult patients undergoing elective colorectal procedures and were required to report on surgical site infection (SSI) within 30 days of surgery.

**Data Extraction and Synthesis:**

Data on SSIs, adverse events, all-cause mortality, and length of hospital stay were extracted when available by 2 independent reviewers. A frequentist random-effects model was used for network meta-analysis. Reporting followed the Preferred Reporting Items for Systematic Review and Meta-Analyses, Extension Statement for Reporting of Systematic Reviews Incorporating Network Meta-Analyses of Health Care Interventions.

**Main Outcomes and Measures:**

The primary outcome was risk of SSI; secondary outcomes were 30-day mortality, adverse events, and length of hospital stay. Risk ratios (RRs) with 95% CIs were calculated for binary outcomes, and mean differences (MDs) with SDs were calculated for continuous outcomes. The certainty of evidence was assessed using the Grading of Recommendations Assessment, Development, and Evaluation (GRADE) approach.

**Findings:**

A total of 105 randomized clinical trials involving 18 273 patients were included. The network included 32 distinct antibiotic nodes defined by antimicrobial class or combination. High to moderate certainty of evidence indicated that, compared with placebo or no antibiotic, several regimens were associated with reduced the risk of SSI. Regimens including broad-spectrum penicillin (RR, 0.26; 95% CI, 0.16-0.42), third-generation cephalosporins (RR, 0.27; 95% CI, 0.16-0.45), a combination of metronidazole and second-generation cephalosporins (RR, 0.27; 95% CI, 0.17-0.44), and tetracyclines (RR, 0.32; 95% CI, 0.20-0.53) were all associated with significant reductions. The antibiotics associated with a significant decrease in mortality compared with placebo were broad-spectrum penicillin (RR, 0.21; 95% CI, 0.05-0.90) and a combination of fluoroquinolones and penicillins (RR, 0.14; 95% CI, 0.03-0.79). No significant differences were observed between antibiotic classes regarding hospital length of stay or adverse events. The certainty of evidence ranged from very low to moderate across outcomes.

**Conclusions and Relevance:**

In this systematic review and network meta-analysis, several antibiotic regimens were associated with substantially reduced risk of SSIs after elective colorectal surgery compared with placebo. Broad-spectrum penicillins had the most consistent benefits, with moderate-certainty evidence supporting reductions in risk of both SSI and mortality.

## Key Points

**Question:**

Which antibiotic prophylaxis regimens are associated with the lowest risk of surgical site infections (SSIs) among patients undergoing elective colorectal surgery?

**Findings:**

In this systematic review and network meta-analysis of 105 randomized clinical trials with 18 273 patients, broad-spectrum penicillins and cephalosporin-based regimens had the strongest associations with reduced risk of SSIs. Regimens including broad-spectrum penicillins were also associated with decreased risk of mortality, but there were no significant differences among regimens with regard to hospital length of stay or adverse events.

**Meaning:**

In this study, broad-spectrum penicillins and cephalosporin-based regimens were most strongly associated with SSI prevention, highlighting antibiotic class selection as a key factor in colorectal surgery prophylaxis.

## Teaser

This systematic review and network meta-analysis uses data from randomized clinical trials to compare the association of different antibiotic classes and class combinations with clinical and safety outcomes, including surgical-site infection, mortality, adverse events, and length of stay among patients undergoing elective colorectal surgery.

## Introduction

Surgical site infections (SSIs) represent one of the most frequent complications after elective colorectal surgery, with an incidence estimated to range from 10% to 25% depending on patient, procedural, and institutional factors.^1,2^ SSIs contribute a greater morbidity, extended hospital stay, and increased antimicrobial use outcomes that are particularly concerning in the context of global antimicrobial resistance and resource stewardship.^3,4^ Optimizing perioperative antibiotic prophylaxis in this high-risk setting is therefore a critical component of both clinical care and infection prevention policy.^5^

The colonic environment presents a unique microbial challenge due to its high burden of aerobic and anaerobic flora. Most international guidelines recommend dual-agent systemic prophylaxis, typically consisting of a cephalosporin in combination with metronidazole, administered intravenously shortly before incision.^6,7,8^ However, there remains considerable heterogeneity in clinical practice, with variation in antibiotic selection, route of administration, and use of adjunctive oral agents. Some regimens rely on β-lactam or β-lactamase inhibitor monotherapy, while others incorporate oral antibiotics, often as part of bowel preparation protocols that differ in timing and composition.^9,10^

Previous meta-analyses have demonstrated the general efficacy of antibiotic prophylaxis in colorectal surgery but have not adequately distinguished between antibiotic classes or standardized administration timing.^11,12,13,14,15^ Moreover, few studies have systematically evaluated the certainty of evidence to inform the strength of resulting clinical inferences.^6,16^

To overcome prior methodological limitations and evidence gaps, we conducted a comprehensive systematic review and network meta-analysis (NMA) of randomized clinical trials (RCTs) assessing antibiotic prophylaxis administered within 24 hours before elective colorectal surgery. Importantly, we applied the Grading of Recommendations Assessment, Development, and Evaluation (GRADE) approach to evaluate the certainty of evidence across all key comparisons, ensuring a transparent appraisal of both effect estimates and their reliability.^17^

## Methods

### Study Design

This review was registered in PROSPERO (CRD42023434544) and conducted in accordance with Preferred Reporting Items for Systematic Reviews and Meta-Analysis, Extension Statement for Reporting of Systematic Reviews Incorporating Network Meta-Analyses of Health Care Interventions (PRISMA-NMA), based on a published protocol.^18,19^ Our objective was to compare the outcomes associated with prophylactic antibiotic regimens administered within 24 hours before elective colorectal surgery, with a focus on both efficacy and safety.

### Eligibility Criteria

We included parallel-group RCTs involving adult patients (≥18 years) undergoing elective colorectal surgery. Eligible studies compared antibiotic regimens administered within 24 hours before incision and reported on at least one of the following outcomes: SSI within 30 days of surgery, 30-day all-cause mortality, any adverse events, or length of hospital stay. Interventions were classified by antibiotic class or combinations of classes, regardless of route of administration. Studies were excluded if they used oral antibiotics initiated more than 24 hours before surgery as part of mechanical bowel preparation (MBP) protocol, enrolled pediatric population, evaluated nonantibiotic interventions (eg, antiseptics, mechanical strategies), failed to report SSI outcomes, compared identical antibiotic classes, or had sample sizes of fewer than 10 patients per group.

### Search Strategy and Study Selection

A comprehensive search of MEDLINE, Embase, Cochrane CENTRAL, CINAHL, and Scopus was developed by a medical librarian and performed from inception through July 17, 2025. The complete electronic search strategy for each database is provided in the eAppendix in Supplement 1. Searches were limited to English-language publications. We used controlled vocabulary and free-text combinations for *colorectal surgery*, *antibiotic prophylaxis*, and *randomized controlled trials*. Additional eligible studies were identified through screening the reference lists of relevant reviews and included articles. Pairs of reviewers (S.M., S.G.K., F.M., F.M.F., T.A.B., and S.K.) independently screened titles, abstracts, and full texts for inclusion, resolving disagreements through consensus.

### Data Extraction

Pairs of reviewers (S.M., S.G.K., F.M., F.M.F., T.A.B., S.I., S.M.F.L., and S.K.) independently extracted data using a standardized extraction form. Extracted variables included study characteristics, patient population, surgical indication, antibiotic regimen (class, dose, timing, and route of administration), and outcome events.

SSIs, the primary outcome, were categorized according to standardized definitions when available: superficial incisional SSI (infection involving only the skin or subcutaneous tissue), deep incisional SSI (infection involving the fascial or muscular layers), and organ or space SSI (infection involving any anatomic space, other than the incision or musculocutaneous layers, that was opened or manipulated during the procedure). When subtype-specific data were reported, these were aggregated to construct an overall SSI measure. Most trials assessed and reported SSIs, all-cause mortality, and adverse events within 30 days postoperatively, although follow-up intervals varied across studies; this variability was accounted for in the GRADE evaluation through judgments related to indirectness and risk of bias. The secondary outcomes (ie, mortality, adverse events, and length of hospital stay) were extracted as defined in each trial, with heterogeneity in outcome definitions and assessment periods reflected in the certainty-of-evidence ratings.

### Risk-of-Bias Assessment

Risk of bias was assessed using a modified Cochrane Risk of Bias tool (RoB 1.0) for randomized trials across 5 domains related to random sequence generation; allocation concealment; blinding of participants, health care practitioners, data collectors, and data adjudicators; and incomplete outcome data (>20% missing data was considered as high risk of bias).^20,21^ We used a modified version with the following answers: definitely yes or probably yes (considered low risk of bias), or definitely no or probably no (considered high risk of bias), rather than the standard responses (high, low, or unclear).^20^ This approach ensured that our risk-of-bias assessments did not rely on an unclear response option and helps reduce satisficing. Disagreements were first resolved through discussion, and if unresolved, adjudicated by a third reviewer (S.M.). Visualization of the risk of bias was assessed using the Robvis tool.^22^

### Certainty of Evidence

The certainty of evidence for all estimates was assessed using the Confidence in Network Meta-Analysis framework, which is endorsed by the Cochrane Collaboration.^23,24^ This approach evaluates 6 key domains. Domains included within-study risk of bias, across-studies risk of bias, indirectness, imprecision, heterogeneity, and incoherence to generate an overall judgment on confidence in each network estimate. Each comparison was rated as high, moderate, low, or very low certainty. Summary of findings tables were generated to support transparency and facilitate interpretation for decision-making.

### Statistical Analysis

Outcomes were summarized using relative risk (RR) and associated 95% CIs for binary outcomes and mean differences (MDs) and SDs for continuous outcomes. We applied established methods, including those outlined in the Cochrane Handbook^21^ and by Hozo et al,^25^ to estimate SDs when they were not directly reported.

A frequentist random-effect NMA was conducted using the Network suite in Stata version 18 (StataCorp).^26,27^ For direct (pairwise) meta-analyses, we additionally applied a DerSimonian-Laird random-effects model, and these direct estimates were used to inform GRADE certainty assessments.

We examined global coherence within each network using the design-by-treatment interaction model (global test)^28^ and assessed local incoherence within closed loops through the side-splitting approach.^29,30^ When data permitted, we explored the potential association of key prognostic factors with treatment effect through network meta-regression. Planned subgroup analysis included: (1) patients with vs without cancer, (2) sex (male vs female), (3) MBP vs no MBP, and (4) overall trial risk of bias (low or moderate vs high).

Interventions were grouped into nodes by antibiotic class or class combinations. All analyses were performed in Stata version 18 (StataCorp). *P* < .05 was considered statistically significant.

## Results

### Study Selection

From 2154 unique citations, 105 RCTs^31,32,33,34,35,36,37,38,39,40,41,42,43,44,45,46,47,48,49,50,51,52,53,54,55,56,57,58,59,60,61,62,63,64,65,66,67,68,69,70,71,72,73,74,75,76,77,78,79,80,81,82,83,84,85,86,87,88,89,90,91,92,93,94,95,96,97,98,99,100,101,102,103,104,105,106,107,108,109,110,111,112,113,114,115,116,117,118,119,120,121,122,123,124,125,126,127,128,129,130,131,132,133,134,135^ enrolling 18 273 patients met eligibility criteria for the network meta-analysis (eFigure in Supplement 1). At the full-text screening stage, 22 studies were excluded because antibiotics were administered more than 24 hours before surgery, 25 trials compared interventions using the same antibiotic class, and 5 used antibiotics that are no longer available in clinical practice. Characteristics of included studies are presented in eTable 1 in Supplement 1.

### Study Characteristics

A total of 32 distinct antibiotic regimens were evaluated across the included trials. These comprised monotherapy with cephalosporins, penicillins, or metronidazole; dual-agent regimens combining cephalosporins or penicillins with metronidazole; and broad-spectrum β-lactamase inhibitors, including ampicillin-sulbactam, amoxicillin-clavulanate, ticarcillin-clavulanate, and mezlocillin (eTable 1 in Supplement 1). The complete list of antibiotics included in each node is provided in the supplement (eTable 2 in Supplement 1). The median sample size across trials was 52 (range, 11-499). The median for the mean age of the participants was 63.5 years (range 18-93).

Most trials^31,32,33,34,35,36,37,38,39,40,41,42,43,44,45,46,47,48,49,50,51,52,53,54,55,56,57,58,59,61,62,63,66,67,68,69,70,71,72,73,74,75,76,77,78,80,81,82,83,84,85,86,87,88,89,90,91,92,93,94,95,96,97,98,99,100,101,102,103,104,105,106,107,108,109,110,111,112,114,116,117,118,119,120,121,122,123,124,125,126,127,128,129,130,131,132,133,134,135^ involved elective colon or rectal resections, with a minority^60,65,79,113,115^ specifying laparoscopic vs open procedures. Eighty studies^31,32,33,36,37,38,40,41,42,43,45,46,48,49,56,58,61,63,64,65,66,67,68,69,70,71,72,74,75,76,77,78,79,80,82,83,84,85,86,88,89,90,91,92,93,94,95,96,97,98,99,100,103,104,106,107,108,109,111,112,117,119,120,121,122,123,124,125,128,129,130,132,133,135^ included a mix of patients with and without cancer, while 7 studies^39,73,101,102,105,110,113^ included only patients with cancer, and 5 studies^34,44,47,57,81^ exclusively enrolled patients without cancer. The use of MBP was explicitly reported conducted in 86 trials (83%),^31,32,33,34,35,37,38,39,40,41,42,43,44,45,46,47,48,49,50,51,52,53,54,55,57,58,59,61,62,63,64,65,66,67,68,69,70,71,73,74,75,76,77,78,79,80,81,83,85,87,88,89,90,91,92,93,95,96,100,101,102,103,104,105,106,107,109,110,111,112,113,117,118,120,121,122,124,125,126,127,128,130,131,132,133,135^ while 10 trials (9.5%)^36,72,82,84,94,98,99,108,123,134^ did not specify bowel preparation status, and the rest reported no MBP.^56,60,86,97,114,115,116,119,129^ The median follow-up duration for SSI outcome assessment was 30 days (range, 14-56), consistent with standard definitions.

### Risk of Bias

Most trials (79 [75.2%])^31,32,33,35,38,39,40,41,42,43,44,45,46,47,48,49,50,51,52,53,54,55,56,58,59,61,63,66,67,68,69,70,72,73,74,76,77,78,81,82,83,84,89,90,94,95,96,97,98,99,100,101,102,103,105,107,110,113,114,115,116,117,118,119,120,121,122,123,124,125,126,127,129,130,131,132,133,135^ were judged to be at high risk of bias in at least 1 domain. Random sequence generation was adequately reported in 91 trials (86.7%),^31,32,33,34,35,36,37,38,40,41,42,43,44,45,46,47,48,50,52,56,57,58,59,60,62,63,64,65,67,68,69,70,71,72,73,74,75,76,77,79,80,81,82,83,84,85,86,87,88,90,91,92,93,94,95,96,97,98,101,102,103,104,106,107,108,109,110,111,112,113,114,115,116,117,118,119,120,121,122,123,124,125,126,128,129,130,131,132,133,134,135^ while allocation concealment was clearly described in only 37 trials (35.2%).^31,34,35,36,37,40,41,42,57,58,60,65,67,68,69,71,73,74,75,76,80,82,84,85,86,87,91,92,93,95,112,114,115,117,120,121,124^ Blinding of participants was performed in 36 trials (34.2%),^32,34,36,37,42,53,54,55,56,57,58,60,61,62,64,65,79,80,85,86,88,91,92,93,102,104,106,108,109,112,124,128,131,132,134,135^ blinding of health care practitioners in 38 trials (36.2%),^32,34,36,37,42,53,54,55,56,57,58,60,61,62,64,65,79,80,85,86,88,91,92,93,102,104,106,108,109,112,124,128,129,130,131,132,134,135^ and blinding of outcome assessors in 34 trials (32.4%).^32,34,36,37,42,53,54,55,56,57,58,60,61,62,64,65,79,80,85,86,88,91,92,93,102,104,106,108,109,112,124,128,132,134^ Most studies (76 [72.5%])^31,32,33,34,36,37,39,40,44,45,47,49,50,51,52,56,60,61,62,63,64,65,66,67,68,70,71,73,74,75,78,79,80,81,82,83,84,85,86,87,88,90,91,92,93,94,95,97,98,99,100,103,104,105,106,107,108,109,110,111,114,115,116,117,118,119,120,121,122,123,124,125,127,128,129,135^ reported fewer than 20% missing outcome data. Overall, 48 trials (44.7%)^33,39,41,43,44,45,46,47,48,49,50,51,52,53,54,55,61,63,66,69,70,72,73,74,76,77,78,81,83,89,97,99,100,101,102,105,107,110,111,113,114,116,118,120,122,123,127,133^ were rated as having a high risk of bias, while 26 (24.8%)^32,34,36,37,57,60,62,64,65,71,75,80,85,86,87,88,91,92,93,104,106,108,109,112,128,134^ were considered to have a low risk of bias (eTable 3 in Supplement 1).

### SSI

All 105 RCTs, including 18 273 patients, reported on SSI within 30 days. Of the 62 direct comparisons included in the network, 25 were informed by more than 1 study. There was no evidence of global (*P* = .12) or loop-specific incoherence (Figure, A).

**Figure.**
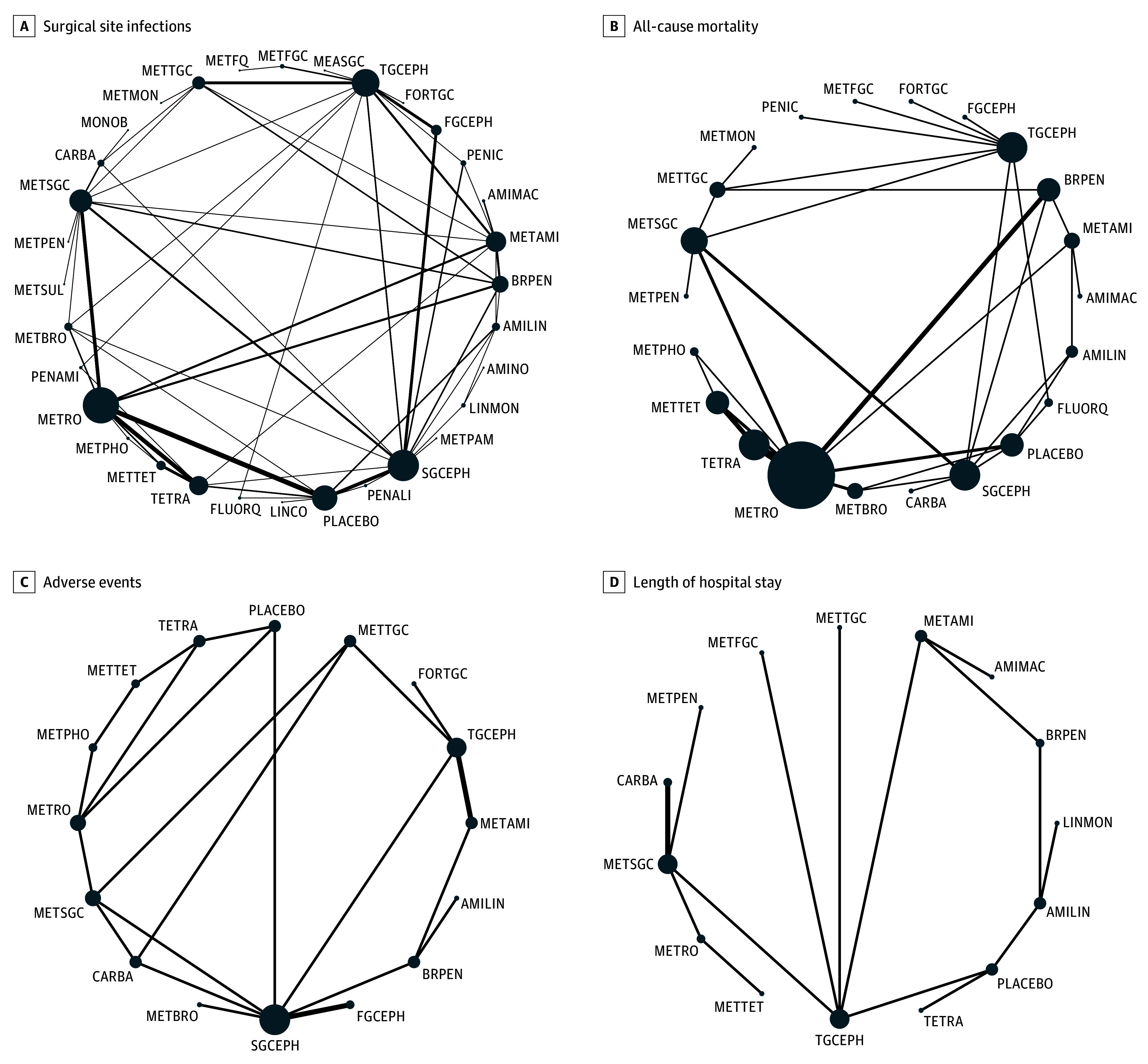
Network of Eligible Comparisons The size of the nodes corresponds to the number of patients randomized to that intervention. The interventions directly compared are linked with a line; the thickness of the line corresponds to the number of trials that assessed the comparison. AMILIN indicates aminoglycosides and lincosamides; AMIMAC, aminoglycosides and macrolides; AMINO, aminoglycosides; BRPEN, broad-spectrum β-lactams; CARBA, carbapenems; FGCEPH, first-generation cephalosporin; FLUORQ, fluoroquinolones; FORTGC, fourth-generation cephalosporin; LINCO, lincosamides; LINMON, lincosamides and monobactams; MEASGC, metronidazole and second-generation cephalosporins and aminoglycosides; MONOB, monobactams; METAMI, aminoglycosides and metronidazole; METBRO, metronidazole and broad-spectrum β-lactams; METFGC, metronidazole and first-generation cephalosporins; METFQ, metronidazole and fluoroquinolones; METMON, metronidazole and monobactams; METPAM, metronidazole, penicillin, and aminoglycoside; METPEN, metronidazole and penicillin; METPHO, metronidazole and phosphonic acid; METRO, metronidazole; METSGC, metronidazole and second-generation cephalosporins; METSUL, metronidazole and sulfonamide; METTET, tetracyclines and metronidazole; METTGC, metronidazole and third-generation cephalosporins; METMON, metronidazole and Monobactam; PENALI, penicillin, aminoglycosides, and lincosamide; PENAMI, penicillin and aminoglycosides; PENIC, penicillin; SGCEPH, second-generation cephalosporins; TETRA, tetracyclines; and TGCEPH, third-generation cephalosporins.

Moderate to high–certainty evidence showed several classes were associated with reduced risk of SSI compared with placebo or no prophylaxis: broad-spectrum penicillins (RR, 0.26; 95% CI, 0.16-0.42), third-generation cephalosporins (RR, 0.27; 95% CI, 0.16-0.43), combination of second-generation cephalosporin plus metronidazole (RR, 0.27; 95% CI, 0.17-0.44), tetracyclines (RR, 0.32; 95% CI, 0.20-0.53), and the combination of metronidazole and aminoglycosides (RR, 0.42; 95% CI, 0.25-0.70) (Table 1; eTable 4 in Supplement 1). Other regimens, such as metronidazole combined with penicillin, metronidazole with a monobactam, or combinations including penicillin, aminoglycosides, and lincosamides were associated with little to no benefit compared with placebo (Table 1; eTable 4 in Supplement 1).

**Table 1.** Network Meta-Analysis Results of Surgical Site Infection Sorted by Certainty of Evidence for Comparisons of Active Interventions vs Placebo

Intervention	RR (95% CI)
**High certainty of evidence^a^**	
Associated with reduced risk	
Broad-spectrum penicillins	0.26 (0.16-0.42)
Third-generation cephalosporins	0.27 (0.16-0.45)
Metronidazole and second-generation cephalosporins	0.27 (0.17-0.44)
Tetracyclines	0.32 (0.20-0.53)
Metronidazole and aminoglycosides	0.42 (0.25-0.70)
Not associated with reduced risk	
Metronidazole and penicillins and aminoglycosides	0.60 (0.18-1.95)
Metronidazole and monobactams	0.71 (0.23-2.20)
Penicillins, aminoglycosides, and lincosamides	0.30 (0.03-3.02)
**Low certainty of evidence** ^b^	
Maybe associated with reduced risk	
Metronidazole and phosphonics	0.07 (0.02-0.25)
Lincosamides and monobactams	0.12 (0.04-0.34)
Metronidazole and tetracyclines	0.20 (0.09-0.42)
Carbapenems	0.24 (0.12-0.45)
Aminoglycosides and lincosamides	0.25 (0.14-0.46)
Maybe less or not associated with reduced risk	
Fourth-generation cephalosporins	0.27 (0.10-0.76)
Metronidazole and broad-spectrum penicillins	0.26 (0.13-0.52)
First-generation cephalosporins	0.27 (0.14-0.52)
Metronidazole and third-generation cephalosporin	0.28 (0.16-0.50)
Second-generation cephalosporins	0.35 (0.22-0.53)
Penicillins	0.40 (0.20-0.81)
Aminoglycosides and macrolides	0.29 (0.10-0.85)
Aminoglycosides	0.19 (0.04-0.86)
Metronidazole	0.46 (0.31-0.67)
Metronidazole and fluoroquinolones	0.20 (0.02-2.19)
Lincosamides	0.32 (0.09-1.10)
Monobactams	0.20 (0.04-1.10)
Metronidazole and first-generation cephalosporin	0.29 (0.07-1.27)
Metronidazole and penicillins	0.46 (0.17-1.24)
Metronidazole and sulfonamides	0.52 (0.19-1.43)
Metronidazole, aminoglycosides, and second-generation cephalosporins	0.54 (0.04-6.55)
Fluoroquinolones	0.62 (0.28-1.33)
Penicillins and aminoglycosides	0.76 (0.17-3.32)

Abbreviation: RR, relative risk.

^a^ Included studies had a high or moderate level of certainty.

^b^ Included studies had a low or very low level of certainty.

Low-certainty evidence showed that 5 antibiotic classes may be associated with reduced risk of SSIs: a combination of metronidazole and fosfomycin (RR, 0.07; 95% CI, 0.02-0.25), lacosamide and monobactams (RR, 0.12; 95% CI 0.04-0.34), metronidazole and tetracyclines (RR, 0.20; 95% CI, 0.09-0.42), carbapenems (RR, 0.24; 95% CI, 0.12-0.45), and combination of aminoglycosides and lacosamide (RR, 0.25; 95% CI, 0.14-0.46) (Table 1; eTable 4 in Supplement 1).

### All-Cause Mortality

Forty-three trials,^31,33,34,42,43,44,45,48,49,50,51,52,53,56,62,64,65,74,80,81,83,87,92,93,98,100,107,110,111,112,113,114,116,118,120,121,122,123,124,125,126,130,135^ including 9219 patients, reported on all-cause mortality. Among the 34 direct comparisons, 8 were informed by at least 2 studies, with no evidence of substantial heterogeneity. Global inconsistency testing indicated no violation of network assumptions (*P* = .99), and loop-specific analyses similarly showed no incoherence (Figure, B). Broad-spectrum penicillins and a combination of fluoroquinolones and penicillins were associated with reduced risk of mortality compared with placebo. For broad-spectrum penicillins, the relative risk was 0.21 (95% CI, 0.05-0.90), supported by a moderate certainty of evidence. A combination of fluoroquinolones and penicillins was also associated with reduced mortality risk (RR, 0.14; 95% CI, 0.03-0.79); the certainty of evidence for this comparison was rated as low (Table 2; eTable 5 in Supplement 1).

**Table 2.** Network Meta-Analysis Results of Mortality Sorted by GRADE Certainty of Evidence for Comparisons of Active Interventions vs Placebo

Intervention	RR (95% CI)
**High certainty of evidence** ^a^	
Associated with reduced risk	
Broad-spectrum penicillins	0.21 (0.05-0.90)
Not associated with reduced risk	
Metronidazole and aminoglycosides	0.32 (0.06-1.66)
Tetracyclines	0.45 (0.09-2.30)
Third-generation cephalosporins	0.37 (0.08-1.69)
Metronidazole and second-generation cephalosporins	0.53 (0.13-2.23)
**Low certainty of evidence** ^b^	
Maybe associated with reduced risk	
Fluoroquinolones and penicillins	0.14 (0.03-0.79)
Maybe not associated with reduced risk	
Aminoglycosides and macrolides	0.08 (0.01-1.28)
Carbapenems	0.21 (0.03-1.47)
Metronidazole and tetracyclines	0.29 (0.05-1.69)
Aminoglycosides and lincosamides	0.33 (0.04-2.66)
Metronidazole and phosphonics	0.30 (0.04-2.28)
Metronidazole and monobactams	0.35 (0.05-2.32)
Fourth-generation cephalosporins	0.28 (0.03-2.34)
Penicillins	0.33 (0.01-7.76)
Metronidazole and third-generation cephalosporin	0.43 (0.10-1.92)
Second-generation cephalosporins	0.48 (0.11-2.02)
Metronidazole	0.61 (0.16-2.35)
Metronidazole and broad-spectrum penicillins	0.63 (0.14-2.80)
Metronidazole and penicillins	1.01 (0.06-16.18)
Metronidazole and first-generation cephalosporin	1.86 (0.14-25.13)
First-generation cephalosporins	7.82 (0.30-203.02)

Abbreviation: RR, relative risk.

^a^ Included studies had a high or moderate level of certainty.

^b^ Included studies had a low or very low level of certainty.

When comparing across active regimens, broad-spectrum penicillins, carbapenems, and a combination of aminoglycosides and macrolides were also associated with lower mortality rate compared with first-generation cephalosporins (eTable 5 in Supplement 1). The certainty of evidence for these comparisons was rated as moderate due to imprecision. All other regimens showed no significant differences, with certainty ranging from low to very low due to imprecision and risk of bias.

### Adverse Events

Twenty-four trials,^32,34,44,45,50,65,67,71,75,79,80,85,86,92,96,103,111,112,113,114,116,117,132,135^ including 6074 patients, reported adverse events, including diarrhea, nausea or vomiting, urticaria, rash, candidiasis, hypotension, phlebitis, and erythema (Figure, C). When pooled across classes, no antibiotic regimen demonstrated a statistically significant difference in risk of adverse events compared with placebo or with other active regimens. The sole exception was the combination of metronidazole plus a broad-spectrum penicillin, which was associated with a significantly lower risk of adverse events compared with metronidazole plus a third-generation cephalosporin (RR, 0.07; 95% CI, 0.01-0.81) (eTable 6 in Supplement 1). The certainty of evidence for adverse event outcomes was rated as moderate to low, with downgrading for risk of bias and imprecision (Table 3).

**Table 3.** Network Meta-Analysis Results for Adverse Events Sorted by Certainty of Evidence for Comparisons of Active Interventions vs Placebo

Intervention	RR (95% CI)
**High certainty of evidence** ^a^	
Broad-spectrum penicillins	2.36 (0.11-49.60)
Metronidazole and second-generation cephalosporins	2.30 (0.16-33.03)
Tetracyclines	1.85 (0.10-33.94)
Third-generation cephalosporins	1.06 (0.04-29.11)
Metronidazole and aminoglycosides	1.22 (0.04-36.94)
**Low certainty of evidence** ^b^	
Metronidazole	3.39 (0.26-43.52)
Metronidazole and third-generation cephalosporin	3.18 (0.20-49.64)
Metronidazole and phosphonics	2.34 (0.06-98.67)
First-generation cephalosporins	2.20 (0.09-54.22)
Metronidazole and tetracyclines	2.20 (0.05-95.92)
Carbapenems	1.52 (0.11-20.96)
Metronidazole and broad-spectrum penicillins	0.23 (0.01-6.79)
Aminoglycosides and lincosamides	0.78 (0.01-64.06)
Fourth-generation cephalosporins	0.87 (0.03-24.39)
Second-generation cephalosporins	0.91 (0.07-12.11)

Abbreviation: RR, relative risk.

^a^ Included studies had a high or moderate level of certainty.

^b^ Included studies had a low or very low level of certainty.

### Length of Hospital Stay

A total of 17 trials,^32,34,44,45,50,65,67,71,75,80,85,86,92,96,103,111,112,113,114,116,117,132,135^ including 2830 patients, reported on the length of hospital stay (Figure, D). Across all comparisons, no antibiotic regimen was associated with hospitalization duration relative to placebo or other active regimens (eTable 7 in Supplement 1). The certainty of evidence for this outcome was rated as moderate to low, downgraded for risk of bias and imprecision (Table 4).

**Table 4.** Network Meta-Analysis Results for Length of Hospital Stay Sorted by Certainty of Evidence for Comparisons of Active Interventions vs Placebo

Intervention	MD (95% CI), d
**High certainty of evidence** ^a^	
Tetracyclines	−7.00 (−19.58 to 5.58)
Broad-spectrum penicillins	−5.79 (−19.80 to 8.22)
Metronidazole and aminoglycosides	−2.85 (−16.96 to 11.26)
Third-generation cephalosporins	−1.52 (−13.41 to 10.38)
Metronidazole and second-generation cephalosporins	0.08 (−17.10 to 17.26)
**Low certainty of evidence** ^b^	
Lincosamides and monobactams	−9.71 (−26.64 to 7.21)
Aminoglycosides and lincosamides	−8.71 (−20.37 to 2.94)
Aminoglycosides and macrolides	−7.85 (−27.40 to 11.71)
Metronidazole and third-generation cephalosporin	−2.52 (−19.72 to 14.69)
Metronidazole and tetracyclines	−2.32 (−27.08 to 22.45)
Metronidazole	1.78 (−19.69 to 23.26)
Metronidazole and first-generation cephalosporin	−1.52 (−18.83 to 15.80)
Carbapenems	−0.89 (−20.25 to 18.47)
Metronidazole and broad-spectrum penicillins	4.08 (−17.21 to 25.38)

Abbreviation: MD, mean difference.

^a^ Included studies had a high or moderate level of certainty.

^b^ Included studies had a low or very low level of certainty.

### Subgroup Analyses

Because event counts were low and data were sparse across many comparisons, most prespecified subgroup analyses could not be undertaken. However, the subgroup analysis based on the overall risk of bias was feasible and did not demonstrate evidence of subgroup effect, with estimates remaining directionally consistent across low- vs moderate- and high-risk studies (eTable 8 in Supplement 1).

## Discussion

This comprehensive NMA of 105 RCTs, including more than 18 000 patients, was conducted to evaluate the comparative outcomes associated with prophylactic antibiotic regimens for elective colorectal surgery. By grouping regimens according to antimicrobial class or class combinations, this analysis provides a more clinically meaningful perspective than prior reviews that focused primarily on route of administration.

Several antibiotic regimens were associated with a significant reduction in the risk of SSI compared with placebo or no prophylaxis. The combination of metronidazole and fosfomycin suggested a possible reduction in SSI risk, although this was supported by low-certainty evidence. Greater confidence was observed for regimens such as broad-spectrum penicillins, third-generation cephalosporins, metronidazole plus second-generation cephalosporins, and tetracyclines, all of which demonstrated moderate certainty of benefit. Notably, broad-spectrum penicillin was the only antibiotic class that was associated with reduced risk of SSI and all-cause mortality.

Previous systematic reviews have mainly examined route-based strategies, such as intravenous (IV) vs oral or combined regimens.^11,13,16,136,137^ The 2020 Cochrane review by Nelson and colleagues^11^ emphasized the superiority of combined oral and IV prophylaxis, especially when paired with concurrent MBP. However, it did not assess the effect of different antibiotic classes.^11^ A 2023 NMA^138^ has supported these findings, reporting benefits from combining oral and IV antibiotics compared with IV alone, but without directly identifying which drug classes confer the greatest protection. Our results expand on this evidence by systematically comparing antimicrobial classes and providing a transparent evaluation of certainty using the GRADE framework. This class-based approach highlights that not all IV regimens are equivalent; certain classes, such as broad-spectrum penicillins, third-generation cephalosporins, and a combination of metronidazole and cephalosporins, appear to be among the most effective.

Importantly, we found no significant differences between regimens in length of hospital stay or rates of adverse events. While this may reflect a genuine absence of a sizeable difference, inconsistent reporting and lack of standardized definitions for adverse outcomes limit definitive conclusions in these domains.

Although many included trials were older, the use of class-based nodes enhances the applicability of our findings to contemporary practice, as antimicrobial classes maintain a similar spectrum and pharmacologic activity across generations. While baseline SSI rates have declined with minimally invasive surgery and modern perioperative care, relative treatment effects remain informative, supporting the relevance of these class-level comparisons to current colorectal surgical practice.

Our study has several strengths, including an extensive literature search across multiple databases; rigorous inclusion criteria focusing on RCTs; a transparent, preregistered protocol; and the assessment of the certainty of evidence using the GRADE approach. The use of NMA allowed integration of direct and indirect evidence across 32 antibiotic nodes, enabling robust comparisons between multiple antibiotic classes, even in the absence of head-to-head trials. Applying the GRADE approach provided a transparent evaluation of the certainty of evidence, an important step rarely undertaken in earlier reviews and essential for translating findings into guidelines and policy.

Our findings reinforce the importance of broad-spectrum penicillins and cephalosporins, especially when combined with anaerobic coverage, as effective measures for preventing SSI in colorectal surgery. The notable effect size seen with metronidazole plus fosfomycin calls for further research, as its clinical use has so far been limited. The potential mortality benefit observed with fluoroquinolones is interesting, but it should be approached with caution due to potential resistance issues and safety warnings associated with this class. Importantly, the lack of significant differences in hospital stays and adverse events across most regimens indicates that preventing SSIs should remain the main factor guiding antibiotic choice in this context.

Although broad-spectrum penicillins and cephalosporin-metronidazole combinations demonstrated favorable comparative effectiveness in our analysis, the choice of prophylactic regimen should be informed by local resistance patterns and patient-specific factors. Individualized strategies based on colonization status or microbiome profiles represent an important future direction but were not evaluated in any of the included trials.

These findings should be interpreted in conjunction with current prophylaxis guidelines for elective colorectal surgery. Existing recommendations from the American Society of Health-System Pharmacists/Infectious Diseases Society of America/Surgical Infection Society/Society for Healthcare Epidemiology of America and the American Society of Colon and Rectal Surgeons emphasize providing both aerobic and anaerobic coverage, rather than prioritizing a specific antibiotic class. Our results complement these guidelines by offering comparative effectiveness data across antibiotic classes that meet these spectrum requirements. Broad-spectrum penicillins and cephalosporin-metronidazole combinations were most consistently associated with reductions in risk of SSI, suggesting that when multiple guideline-endorsed options are available, these regimens may be preferred. Importantly, our mortality and adverse-event findings are intended to support, rather than overturn, current guidance, providing clinicians with additional evidence to refine antibiotic selection within the framework of established recommendations.

Future RCTs should prioritize head-to-head comparisons of leading regimens, including underexplored combinations such as metronidazole plus fosfomycin, in adequately powered, multicenter settings. Improved reporting of patient comorbidities, cancer status, and standardized definitions of adverse events will be essential to refine the evidence base. Finally, given the global challenge of antimicrobial resistance, studies examining the ecological consequences of prophylactic regimens, alongside their efficacy, are urgently needed.

### Limitations

Limitations must also be acknowledged. Despite the large body of evidence, many comparisons were informed by few or small trials, leading to imprecision. Risk of bias was frequent, particularly related to allocation concealment and blinding, which contributed to the downgrading of evidence certainty. Reporting of baseline patient comorbidities, cancer status, and bowel preparation detail was inconsistent, limiting our ability to explore effect modification in subgroup analyses. Another limitation is the variability in how outcomes were defined and assessed across trials. Definitions for SSI, adverse events, mortality, and length of hospital stay were not entirely uniform, and follow-up intervals differed among studies. Although we harmonized data extraction where possible and incorporated this heterogeneity into the GRADE assessments through downgrading for indirectness and risk of bias, these differences may still influence comparability across trials and should be considered when interpreting the findings. Additionally, while our exclusion of oral antibiotic regimens initiated more than 24 hours before surgery ensured focus on prophylaxis, it limits direct comparability with some earlier meta-analyses of bowel preparation strategies.

## Conclusions

This systematic review and NMA of 105 RCTs found that broad-spectrum penicillins and cephalosporin-based combinations had the strongest associations with reduced risk of SSI after elective colorectal surgery, with broad-spectrum penicillins also associated with a mortality reduction benefit. These findings highlight the importance of antibiotic class selection in prophylactic strategies and provide an evidence base to guide future guideline updates and clinical practice.

## Notes

Supplement 1. **eAppendix.** Search Strategy **eFigure.** Preferred Reporting Items for Systematic Review and Meta-Analysis (PRISMA) Flow Diagram for Study Selection **eTable 1.** Summary of Characteristics of 105 Included Studies for Network Meta-Analysis **eTable 2.** Node Definitions for Antibiotic Regimens in the Network Meta-Analysis **eTable 3.** Risk of Bias Assessments for Included Trials **eTable 4.** League Table for Surgical Site Infection—Relative Risk (95% CI) **eTable 5.** League Table for All-Cause Mortality—Relative Risk (95% CI) **eTable 6.** League Table for Adverse Events—Relative Risk (95% CI) **eTable 7.** League Table for Length of Hospital Stay—Mean Difference (95% CI) **eTable 8.** Subgroup Analysis for Surgical Site Infection—Low Risk of Bias vs High Risk of Bias
eReferences.

Supplement 2.
Data Sharing Statement
