# Supplementary material for: Antibiotic Prophylaxis Strategies and Surgical Site Infections in Colorectal Surgery: A Systematic Review and Network Meta-Analysis
Source: JAMA Netw Open. 2026 Feb 19;9(2):e2560095. doi: 10.1001/jamanetworkopen.2025.60095 (PMC12921537; doi:10.1001/jamanetworkopen.2025.60095)
Supplement: Supplement 1. — eAppendix. Search Strategy eFigure. Preferred Reporting Items for Systematic Review and Meta-Analysis (PRISMA) Flow Diagram for Study Selection eTable 1. Summary of Characteristics of 105 Included Studies for Network Meta-Analysis eTable 2. Node Definitions for Antibiotic Regimens in the Network Meta-Analysis eTable 3. Risk of Bias Assessments for Included Trials eTable 4. League Table for Surgical Site Infection—Relative Risk (95% CI) eTable 5. League Table for All-Cause Mortality—Relative Risk (95% CI) eTable 6. League Table for Adverse Events—Relative Risk (95% CI) eTable 7. League Table for Length of Hospital Stay—Mean Difference (95% CI) eTable 8. Subgroup Analysis for Surgical Site Infection—Low Risk of Bias vs High Risk of Bias eReferences. [file jamanetwopen-e2560095-s001.pdf]

Supplemental Online Content

Motaghi S, Karam SG, Mulazzani F, et al. Antibiotic prophylaxis strategies and surgical site infections in colorectal surgery. *JAMA Netw Open.* 2026;9(2):e2560095. doi:10.1001/jamanetworkopen.2025.60095

eAppendix. Search Strategy

eFigure. Preferred Reporting Items for Systematic Review and Meta-Analysis (PRISMA) Flow Diagram for Study Selection

Table 1. Summary of Characteristics of 105 Included Studies for Network Meta-Analysis

eTable 2. Node Definitions for Antibiotic Regimens in the Network Meta-Analysis

eTable 3. Risk of Bias Assessments for Included Trials

eTable 4. League Table for Surgical Site Infection—Relative Risk (95% CI)

eTable 5. League Table for All-Cause Mortality—Relative Risk (95% CI)

eTable 6. League Table for Adverse Events—Relative Risk (95% CI)

eTable 7. League Table for Length of Hospital Stay—Mean Difference (95% CI)

eTable 8. Subgroup Analysis for Surgical Site Infection—Low Risk of Bias vs High Risk of Bias

eReferences.

This supplemental material has been provided by the authors to give readers additional information about their work.

© 2026 Motaghi S et al. *JAMA Network Open*.

eAppendix. Search Strategy

MEDLINE (OVID)

----

1 colorectal surgery.mp. or colorectal/ (11344)

2 Colectomy/ or colectom*.mp. (22675)

3 or/1-39 (31962)

4 exp Anti-Bacterial Agents/ (528964)

5 Antibiotic Prophylaxis/ (14535)

6 cefuroxime.mp. or Cefuroxime/ (4040)

7 metronidazole.mp. or Metronidazole/ (16623)

8 cefazolin.mp. or Cefazolin/ (4382)

9 levofloxacin.mp. or Levofloxacin/ (11380)

10 clindamycin.mp. or Clindamycin/ (10915)

11 vancomycin.mp. or Vancomycin/ (35920)

12 ciprofloxacin.mp. or Ciprofloxacin/ (32614)

13 Ampicillin/ or ampicillin.mp. (20665)

14 aztreonam.mp. or Aztreonam/ (2974)

15 cefotaxime.mp. or Cefotaxime/ (7701)

16 cefoxitin.mp. or Cefoxitin/ (3293)

17 cefotetan.mp. or Cefotetan/ (284)

18 ceftriaxone.mp. or Ceftriaxone/ (14511)

19 Ertapenem/ or ertapenem.mp. (2385)

20 fluconazole.mp. or Fluconazole/ (15792)

21 gentamicins.mp. or Gentamicins/ (8931)

22 moxifloxacin.mp. or Moxifloxacin/ (6784)

23 Piperacillin, Tazobactam Drug Combination/ or piperacillin.mp. (8339)

24 sultamicillin.mp. (424)

25 sulbactam.mp. or Sulbactam/ (3863)

26 Erythromycin/ or erythromycin.mp. (17806)

27 neomycin.mp. or Neomycin/ (6133)

28 (antibacterial or antibiotic or antimicrobial or antiinfectve or anti bacterial or anti biotic or antimicrobial or anti infective).mp. [mp=title, book title, abstract, original title, name of substance word, subject heading word, floating sub-heading word, keyword heading word, organism supplementary concept word, protocol supplementary concept

© 2026 Motaghi S et al. *JAMA Network Open*.

word, rare disease supplementary concept word, unique identifier, synonyms, population supplementary concept word, anatomy supplementary concept word] (666436)

29 or/41-65 (863776)

30 40 and 66 (1409)

31 randomized controlled trial.pt. (541097)

32 controlled clinical trial.pt. (50400)

33 randomi?ed.ab. (768314)

34 placebo.ab. (215178)

35 drug therapy.fs. (2161168)

36 randomly.ab. (419500)

37 trial.ab. (691196)

38 groups.ab. (2489854)

39 or/68-75 (5235087)

40 exp animals/ not humans.sh. (3110613)

41 76 not 77 (4634104)

42 67 and 78 (643)

Embase (OVID)

----

1 colorectal surgery.mp. or colorectal/ (26092)

2 colectomy/ or colectom*.mp. (49200)

3 or/1-2 (71112)

4 exp antibiotic agent/ (2014177)

5 antibiotic prophylaxis/ (43368)

6 cefuroxime/ or cerufoxime.mp. (30432)

7 metronidazole/ or metronidazole.mp. (90203)

8 cefazolin.mp. or cefazolin/ (39653)

9 levofloxacin/ or levofloxacin.mp. (58076)

10 clindamycin/ or clindamycin.mp. (69733)

11 vancomycin.mp. or vancomycin/ (138499)

12 ciprofloxacin/ or ciprofloxacin.mp. (139270)

13 ampicillin/ or ampicillin.mp. (118132)

14 aztreonam.mp. or aztreonam/ (22920)

15 cefotaxime.mp. or cefotaxime/ (55751)

© 2026 Motaghi S et al. *JAMA Network Open*.

16 cefoxitin.mp. or cefoxitin/ (25677)

17 cefotetan.mp. or cefotetan/ (3792)

18 ceftriaxone/ or ceftriaxone.mp. (92008)

19 ertapenem.mp. or ertapenem/ (12815)

20 fluconazole/ or fluconazole.mp. (59333)

21 gentamicins.mp. or gentamicin/ (138429)

22 moxifloxacin.mp. or moxifloxacin/ (28460)

23 piperacillin.mp. or piperacillin/ (66048)

24 sultamicillin.mp. or sultamicillin/ (16540)

25 sulbactam.mp. or sulbactam/ (15786)

26 erythromycin.mp. or erythromycin/ (94582)

27 neomycin/ or neomycin.mp. (28747)

28 (antibacterial or antibiotic or antimicrobial or antiinfectve or anti bacterial or anti biotic or antimicrobial or anti infective).mp. [mp=title, abstract, heading word, drug trade name, original title, device manufacturer, drug manufacturer, device trade name, keyword heading word, floating subheading word, candidate term word] (1223727)

29 or/4-28 (2436317)

30 3 and 29 (8061)

31 randomized controlled trial/ (876206)

32 Controlled clinical study/ (445322)

33 random$.ti,ab. (2193002)

34 randomization/ (100581)

35 intermethod comparison/ (313617)

36 placebo.ti,ab. (391431)

37 (compare or compared or comparison).ti. (648514)

38 ((evaluated or evaluate or evaluating or assessed or assess) and (compare or compared or comparing or comparison)).ab. (3116441)

39 (open adj label).ti,ab. (123153)

40 ((double or single or doubly or singly) adj (blind or blinded or blindly)).ti,ab. (293211)

41 double blind procedure/ (230826)

42 parallel group$1.ti,ab. (35291)

43 (crossover or cross over).ti,ab. (133355)

44 ((assign$ or match or matched or allocation) adj5 (alternate or group$1 or intervention$1 or patient$1 or subject$1 or participant$1)).ti,ab. (456591)

45 (assigned or allocated).ti,ab. (540012)

© 2026 Motaghi S et al. *JAMA Network Open*.

46 (controlled adj7 (study or design or trial)).ti,ab. (500635)

47 (volunteer or volunteers).ti,ab. (297246)

48 human experiment/ (688475)

49 trial.ti. (454034)

50 or/31-49 (6944326)

51 (random$ adj sampl$ adj7 ("cross section$" or questionnaire$1 or survey$ or database$1)).ti,ab. not (comparative study/ or controlled study/ or randomi?ed controlled.ti,ab. or randomly assigned.ti,ab.) (10388)

52 Cross-sectional study/ not (randomized controlled trial/ or controlled clinical study/ or controlled study/ or randomi?ed controlled.ti,ab. or control group$1.ti,ab.) (436497)

53 (((case adj control$) and random$) not randomi?ed controlled).ti,ab. (23442)

54 (Systematic review not (trial or study)).ti. (320606)

55 (nonrandom$ not random$).ti,ab. (20108)

56 "Random field$".ti,ab. (3143)

57 (random cluster adj3 sampl$).ti,ab. (1746)

58 (review.ab. and review.pt.) not trial.ti. (1274929)

59 "we searched".ab. and (review.ti. or review.pt.) (57526)

60 "update review".ab. (153)

61 (databases adj4 searched).ab. (75448)

62 (rat or rats or mouse or mice or swine or porcine or murine or sheep or lambs or pigs or piglets or rabbit or rabbits or cat or cats or dog or dogs or cattle or bovine or monkey or monkeys or trout or marmoset$1).ti. and animal experiment/ (1295936)

63 Animal experiment/ not (human experiment/ or human/) (2731598)

64 or/51-63 (4788495)

65 50 not 64 (6099824)

66 30 and 65 (1548)

Cochrane Library (Wiley)

ID	Search	Hits
#1	(colectom*):ti,ab,kw (Word variations have been searched)	2061
#2	MeSH descriptor: [Colectomy] explode all trees	1048
#3	#1 or #2	2186
#4	MeSH descriptor: [Anti-Bacterial Agents] explode all trees	16784
#5	MeSH descriptor: [Antibiotic Prophylaxis] explode all trees	1740
#6	MeSH descriptor: [Cefuroxime] explode all trees	535

© 2026 Motaghi S et al. *JAMA Network Open*.

#7 MeSH descriptor: [Metronidazole] explode all trees 2743

#8 MeSH descriptor: [Cefazolin] explode all trees 651

#9 MeSH descriptor: [Levofloxacin] explode all trees 788

#10 MeSH descriptor: [Clindamycin] explode all trees 1054

#11 MeSH descriptor: [Vancomycin] explode all trees 1059

#12 MeSH descriptor: [Ciprofloxacin] explode all trees 1411

#13 MeSH descriptor: [Metronidazole] explode all trees 2743

#14 MeSH descriptor: [Cefazolin] explode all trees 651

#15 MeSH descriptor: [Levofloxacin] explode all trees 788

#16 MeSH descriptor: [Clindamycin] explode all trees 1054

#17 MeSH descriptor: [Vancomycin] explode all trees 1059

#18 MeSH descriptor: [Ciprofloxacin] explode all trees 1411

#18 MeSH descriptor: [Ampicillin] explode all trees 5096

#20 MeSH descriptor: [Aztreonam] explode all trees 210

#21 MeSH descriptor: [Cefotaxime] explode all trees 1659

#22 MeSH descriptor: [Cefoxitin] explode all trees 342

#23 MeSH descriptor: [Cefotetan] explode all trees 126

#24 MeSH descriptor: [Ceftriaxone] explode all trees 863

#25 MeSH descriptor: [Ertapenem] explode all trees 146

#26 MeSH descriptor: [Fluconazole] explode all trees 753

#27 MeSH descriptor: [Gentamicins] explode all trees 1403

#28 MeSH descriptor: [Moxifloxacin] explode all trees 1066

#29 MeSH descriptor: [Piperacillin] explode all trees 522

#30 MeSH descriptor: [Sulbactam] explode all trees 264

#31 MeSH descriptor: [Erythromycin] explode all trees 4300

#32 MeSH descriptor: [Neomycin] explode all trees 432

#33 (Cefuroxime or metronidazole or cefazolin or levofloxacin or clindamycin or vancomycin or ciprofloxacin or ampicillin or aztreonam or cefotaxime or cefoxitin or cefotetan or ceftriaxone or ertapenem or fluconazole or gentamicins or moxifloxacin or piperacillin or sultamicillin or sulbactam or erythromycin or neomycin):ti,ab,kw (Word variations have been searched) 26068

#34 (antibacterial or antibiotic or antimicrobial or antiinfectve or anti bacterial or anti biotic or antimicrobial or anti infective):ti,ab,kw (Word variations have been searched) 79462

#35 #4 or #5 or #6 or #7 or #8 or #9 or #10 or #11 or #12 or #13 or #14 or #15 or #16 or #17 or #18 or #19 or #20 or #21 or #22 or #23 or #24 or #25 or #26 or #27 or #28 or #29 or #30 or #31 or #32 or #33 or #34 92287

#36 #3 AND #35 in Trials 232

© 2026 Motaghi S et al. *JAMA Network Open*.

CINAHL (EBSCO)

#	Query	Limiters/Expanders	Last Run Via	Results
S91	S67 AND S90	Search modes - Boolean/Phrase	Interface - EBSCOhost Research Databases<br>Search Screen - Advanced Search<br>Database - CINAHL	845
S90	S89 NOT S88	Expanders - Apply equivalent subjects<br>Search modes - Boolean/Phrase	Interface - EBSCOhost Research Databases<br>Search Screen - Advanced Search<br>Database - CINAHL	955,293
S89	S68 OR S69 OR S70 OR S71 OR S72 OR S73 OR S74 OR S75 OR S76 OR S77 OR S78 OR S79 OR S80 OR S81 OR S82	Expanders - Apply equivalent subjects<br>Search modes - Boolean/Phrase	Interface - EBSCOhost Research Databases<br>Search Screen - Advanced Search<br>Database - CINAHL	1,002,385
S88	S86 NOT S87	Expanders - Apply equivalent subjects<br>Search modes - Boolean/Phrase	Interface - EBSCOhost Research Databases<br>Search Screen - Advanced Search<br>Database - CINAHL	212,330
S87	MH (human)	Expanders - Apply equivalent subjects<br>Search modes - Boolean/Phrase	Interface - EBSCOhost Research Databases<br>Search Screen - Advanced Search<br>Database - CINAHL	2,655,995
S86	S83 OR S84 OR S85	Expanders - Apply equivalent subjects<br>Search modes - Boolean/Phrase	Interface - EBSCOhost Research Databases<br>Search Screen - Advanced Search<br>Database - CINAHL	246,038
S85	TI (animal model*)	Expanders - Apply equivalent subjects<br>Search modes - Boolean/Phrase	Interface - EBSCOhost Research Databases<br>Search Screen - Advanced Search<br>Database - CINAHL	3,476
S84	MH (animal studies)	Expanders - Apply equivalent subjects<br>Search modes - Boolean/Phrase	Interface - EBSCOhost Research Databases<br>Search Screen - Advanced Search<br>Database - CINAHL	151,273
S83	MH animals+	Expanders - Apply equivalent subjects<br>Search modes - Boolean/Phrase	Interface - EBSCOhost Research Databases<br>Search Screen - Advanced Search<br>Database - CINAHL	103,722
S82	AB (cluster W3 RCT)	Expanders - Apply equivalent subjects<br>Search modes - Boolean/Phrase	Interface - EBSCOhost Research Databases<br>Search Screen - Advanced Search<br>Database - CINAHL	487

© 2026 Motaghi S et al. *JAMA Network Open*.

S81	MH (crossover design) OR MH (comparative studies)	Expanders - Apply equivalent subjects Search modes - Boolean/Phrase	Interface - EBSCOhost Research Databases Search Screen - Advanced Search Database - CINAHL	470,857
S80	AB (control W5 group)	Expanders - Apply equivalent subjects Search modes - Boolean/Phrase	Interface - EBSCOhost Research Databases Search Screen - Advanced Search Database - CINAHL	142,719
S79	PT (randomized controlled trial)	Expanders - Apply equivalent subjects Search modes - Boolean/Phrase	Interface - EBSCOhost Research Databases Search Screen - Advanced Search Database - CINAHL	149,762
S78	MH (placebos)	Expanders - Apply equivalent subjects Search modes - Boolean/Phrase	Interface - EBSCOhost Research Databases Search Screen - Advanced Search Database - CINAHL	13,652
S77	MH (sample size) AND AB (assigned OR allocated OR control)	Expanders - Apply equivalent subjects Search modes - Boolean/Phrase	Interface - EBSCOhost Research Databases Search Screen - Advanced Search Database - CINAHL	4,454
S76	TI (trial)	Expanders - Apply equivalent subjects Search modes - Boolean/Phrase	Interface - EBSCOhost Research Databases Search Screen - Advanced Search Database - CINAHL	177,662
S75	AB (random*)	Expanders - Apply equivalent subjects Search modes - Boolean/Phrase	Interface - EBSCOhost Research Databases Search Screen - Advanced Search Database - CINAHL	395,045
S74	TI (randomised OR randomized)	Expanders - Apply equivalent subjects Search modes - Boolean/Phrase	Interface - EBSCOhost Research Databases Search Screen - Advanced Search Database - CINAHL	138,134
S73	MH cluster sample	Expanders - Apply equivalent subjects Search modes - Boolean/Phrase	Interface - EBSCOhost Research Databases Search Screen - Advanced Search Database - CINAHL	5,225
S72	MH pretest-posttest design	Expanders - Apply equivalent subjects Search modes - Boolean/Phrase	Interface - EBSCOhost Research Databases Search Screen - Advanced Search Database - CINAHL	52,291
S71	MH random assignment	Expanders - Apply equivalent subjects Search modes - Boolean/Phrase	Interface - EBSCOhost Research Databases Search Screen - Advanced Search Database - CINAHL	78,215

© 2026 Motaghi S et al. *JAMA Network Open*.

S70	MH single-blind studies	Expanders - Apply equivalent subjects Search modes - Boolean/Phrase	Interface - EBSCOhost Research Databases Search Screen - Advanced Search Database - CINAHL	15,947
S69	MH double-blind studies	Expanders - Apply equivalent subjects Search modes - Boolean/Phrase	Interface - EBSCOhost Research Databases Search Screen - Advanced Search Database - CINAHL	53,983
S68	MH randomized controlled trials	Expanders - Apply equivalent subjects Search modes - Boolean/Phrase	Interface - EBSCOhost Research Databases Search Screen - Advanced Search Database - CINAHL	136,556
S67	S40 AND S66	Search modes - Boolean/Phrase	Interface - EBSCOhost Research Databases Search Screen - Advanced Search Database - CINAHL	146
S66	S41 OR S42 OR S43 OR S44 OR S45 OR S46 OR S47 OR S48 OR S49 OR S50 OR S51 OR S52 OR S53 OR S54 OR S55 OR S56 OR S57 OR S58 OR S59 OR S60 OR S61 OR S62 OR S63 OR S64 OR S65	Search modes - Boolean/Phrase	Interface - EBSCOhost Research Databases Search Screen - Advanced Search Database - CINAHL	153,929
S65	TX antibacterial or antibiotic or antimicrobial or antiinfectve or anti bacterial or anti biotic or antimicrobial or anti infective	Search modes - Boolean/Phrase	Interface - EBSCOhost Research Databases Search Screen - Advanced Search Database - CINAHL	122,193
S64	"neomycin"	Search modes - Boolean/Phrase	Interface - EBSCOhost Research Databases Search Screen - Advanced Search Database - CINAHL	458
S63	(MH "Erythromycin") OR "erythromycin"	Search modes - Boolean/Phrase	Interface - EBSCOhost Research Databases Search Screen - Advanced Search Database - CINAHL	2,188
S62	"sulbactam"	Search modes - Boolean/Phrase	Interface - EBSCOhost Research Databases Search Screen - Advanced Search Database - CINAHL	422
S61	"sultamicillin"	Search modes - Boolean/Phrase	Interface - EBSCOhost Research Databases Search Screen - Advanced Search Database - CINAHL	4
S60	"piperacillin"	Search modes - Boolean/Phrase	Interface - EBSCOhost Research Databases Search Screen - Advanced Search Database - CINAHL	1,267

© 2026 Motaghi S et al. *JAMA Network Open*.

S59	"moxifloxacin"	Search modes - Boolean/Phrase	Interface - EBSCOhost Research Databases<br>Search Screen - Advanced Search<br>Database - CINAHL	1,033
S58	(MH "Gentamicins") OR "gentamicins"	Search modes - Boolean/Phrase	Interface - EBSCOhost Research Databases<br>Search Screen - Advanced Search<br>Database - CINAHL	1,848
S57	(MH "Fluconazole") OR "fluconazole"	Search modes - Boolean/Phrase	Interface - EBSCOhost Research Databases<br>Search Screen - Advanced Search<br>Database - CINAHL	2,396
S56	(MH "Ertapenem") OR "ertapenem"	Search modes - Boolean/Phrase	Interface - EBSCOhost Research Databases<br>Search Screen - Advanced Search<br>Database - CINAHL	382
S55	(MH "Ceftriaxone") OR "ceftriaxone"	Search modes - Boolean/Phrase	Interface - EBSCOhost Research Databases<br>Search Screen - Advanced Search<br>Database - CINAHL	3,050
S54	(MH "Cefotetan") OR "cefotetan"	Search modes - Boolean/Phrase	Interface - EBSCOhost Research Databases<br>Search Screen - Advanced Search<br>Database - CINAHL	55
S53	(MH "Cefoxitin") OR "cefoxitin"	Search modes - Boolean/Phrase	Interface - EBSCOhost Research Databases<br>Search Screen - Advanced Search<br>Database - CINAHL	311
S52	(MH "Cefotaxime") OR "cefotaxime"	Search modes - Boolean/Phrase	Interface - EBSCOhost Research Databases<br>Search Screen - Advanced Search<br>Database - CINAHL	974
S51	(MH "Aztreonam") OR "aztreonam"	Search modes - Boolean/Phrase	Interface - EBSCOhost Research Databases<br>Search Screen - Advanced Search<br>Database - CINAHL	393
S50	"ampicillin"	Search modes - Boolean/Phrase	Interface - EBSCOhost Research Databases<br>Search Screen - Advanced Search<br>Database - CINAHL	2,587
S49	"ciprofloxacin"	Search modes - Boolean/Phrase	Interface - EBSCOhost Research Databases<br>Search Screen - Advanced Search<br>Database - CINAHL	4,077
S48	(MH "Vancomycin") OR "vancomycin"	Search modes - Boolean/Phrase	Interface - EBSCOhost Research Databases<br>Search Screen - Advanced Search<br>Database - CINAHL	7,516
S47	"clindamycin"	Search modes - Boolean/Phrase	Interface - EBSCOhost Research Databases<br>Search Screen - Advanced Search<br>Database - CINAHL	2,144
S46	"levofloxacin"	Search modes - Boolean/Phrase	Interface - EBSCOhost Research Databases<br>Search Screen - Advanced Search<br>Database - CINAHL	1,378

© 2026 Motaghi S et al. *JAMA Network Open*.

S45	(MH "Cefazolin") OR "cefazolin"	Search modes - Boolean/Phrase	Interface - EBSCOhost Research Databases<br>Search Screen - Advanced Search<br>Database - CINAHL	1,004
S44	(MH "Metronidazole") OR "metronidazole"	Search modes - Boolean/Phrase	Interface - EBSCOhost Research Databases<br>Search Screen - Advanced Search<br>Database - CINAHL	3,253
S43	(MH "Cefuroxime") OR "cefuroxime"	Search modes - Boolean/Phrase	Interface - EBSCOhost Research Databases<br>Search Screen - Advanced Search<br>Database - CINAHL	718
S42	(MH "Antibiotic Prophylaxis")	Search modes - Boolean/Phrase	Interface - EBSCOhost Research Databases<br>Search Screen - Advanced Search<br>Database - CINAHL	6,415
S41	(MH "Antibiotics+")	Search modes - Boolean/Phrase	Interface - EBSCOhost Research Databases<br>Search Screen - Advanced Search<br>Database - CINAHL	89,510
S3	(MH "Colectomy") OR "colectom*"	Search modes - Boolean/Phrase	Interface - EBSCOhost Research Databases<br>Search Screen - Advanced Search<br>Database - CINAHL	Display

Scopus (Clarivate)

(((TITLE-ABS-KEY ("colorectal surgery" OR "colon surgery" OR "rectal surgery" OR "colorectal resection" OR "colon resection" OR "rectal resection" OR "colorectal procedure*" OR "colorectal operation*" OR "colorectal surgeon*")) AND (TITLE (rct OR trial OR randomized OR randomised OR placebo))) AND ((TITLE-ABS-KEY (antibacterial OR antibiotic OR antimicrobial OR antiinfectve OR anti AND bacterial OR anti AND biotic OR antimicrobial OR anti AND infective)) OR (TITLE-ABS-KEY (cefuroxime OR metronidazole OR cefazolin OR levofloxacin OR clindamycin OR vancomycin OR ciprofloxacin OR ampicillin OR aztreonam OR cefotaxime OR cefoxitin OR cefotetan OR ceftriaxone OR ertapenem OR fluconazole OR gentamicins OR moxifloxacin OR piperacillin OR sultamicillin OR sulbactam OR erythromycin OR neomycin)))

© 2026 Motaghi S et al. *JAMA Network Open*.

eFigure. Preferred Reporting Items for Systematic Review and Meta-Analysis (PRISMA) Flow Diagram for Study Selection

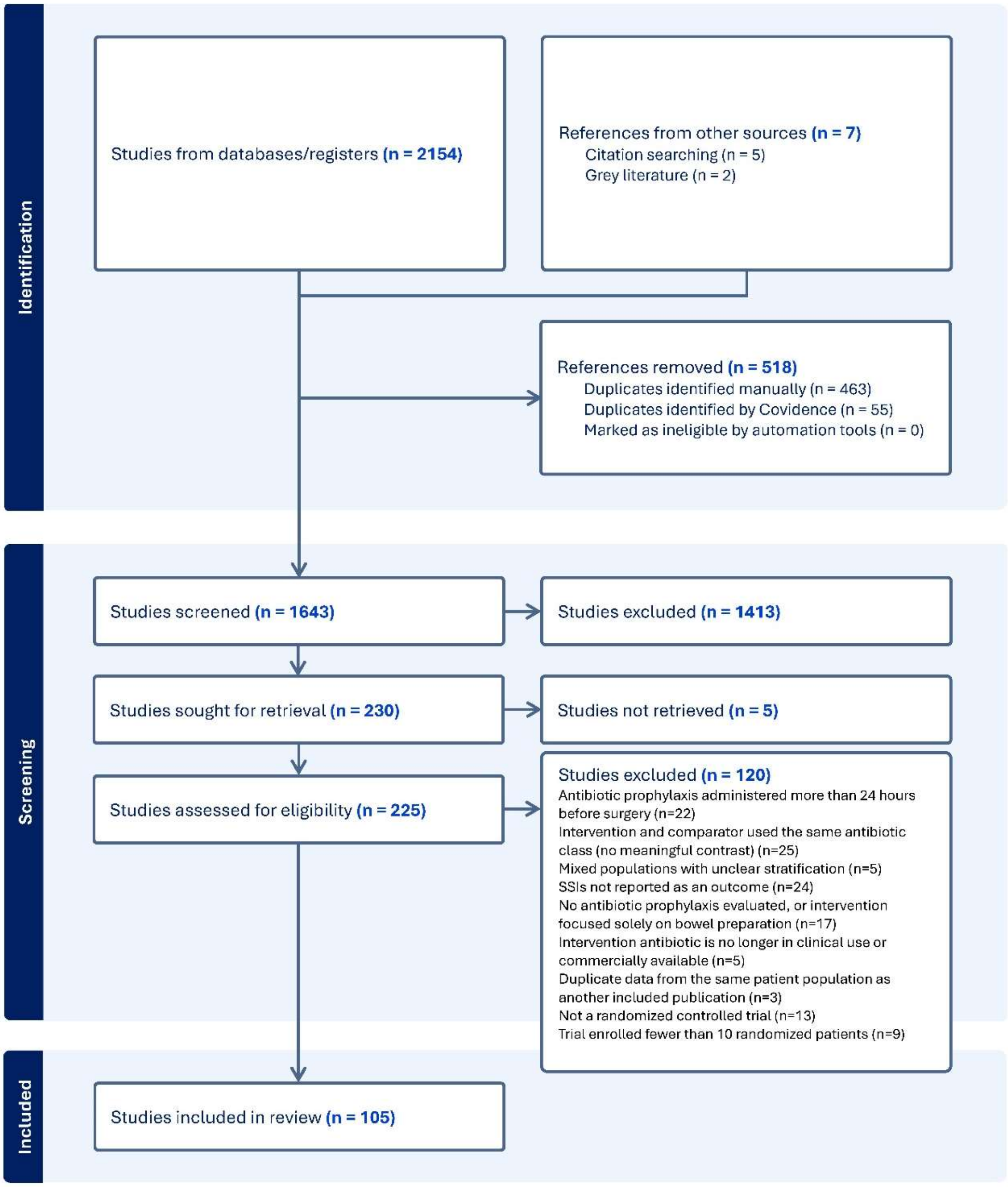

© 2026 Motaghi S et al. *JAMA Network Open*.

Table 1. Summary of Characteristics of 105 Included Studies for Network Meta-Analysis

Author, Year	% with cancer	Age	SD	Percent Male (%)	Intervention	No. randomized	Comparator	No. randomized
Aberg 1984[1]	84	65.9	10.97	45.85	Metronidazole	81	Doxycycline	76
AhChong 1994[2]	93	63	15.63	57.81	ampicillin/*sulbactam	63	gentamicin/metronidazole	65
Ambrose 1983[3]	73.11	62	15.93	44.79	Mezlocillin/metronidazole	49	Cefuroxime/metronidazole	47
Andåker 1992[4]	NA	66.5	14	47.96	Fosfomycin/metronidazole	259	Doxycycline/metronidazole	258
Antonelli 1985[5]	NA	66.2	12	50.64	cefoxitin	40	cephalothin	37
Arnaud 1992[6]	73%	66	12	55	Amoxycillin/clavulanic acid	105	cefotetan	103
Barber 1979[7]	67%	60	NR	NR	gentamycin+clyndamycin	31	Placebo	29
Bell 1983[8]	56.1	53	NR	49.59	Erythromycin gluceptate+tobramycin	61	Metronidazole/tobramycin	62
Bellantone 1988[9]	100	64.89	NR	55.39	Cefotetan+ metronidazole	36	Clindamycin/aztreonam/metronidazole	29
Blair 1987[10]	33.69	43	(range 15 to 86)	53.26	Ticarcillin/clavulanic Acid (Timentin)	50	metronidazole/netilmicin	49
Brolin 1986[11]	57	66.6	(Range18 to 86)	50	Netilmicin, metronidazole	50	Doxycycline	50
Cainzos 1986[12]	71.67	55	(Range 19 to 82)	65	Gentamicin/clindamycin	30	Cefoxitin	30
Cunliffe 1985[13]	66.25	61.1	13.79	38.75	Metronidazole/ cefuroxime	40	Metronidazole	40
Cuthbertson 1983[14]	NR	62.5	14.47	48.26	TInIdazole/cephamandole	100	Tinidazole	111
de la Hunt 1986[15]	71.73	65.9	NR	48.91	Sulbactam/ampicillin	44	Cefoxitin	48

© 2026 Motaghi S et al. *JAMA Network Open*.

Diamond 1988[16]	70.19	60.7	17.82	36.53	mezlocillin	51	Cefuroxime/metronidazole	53
Eykyn 1979[17]	NR	61	NR	48.19	Metronidazole	44	Placebo	39
Favre 1984[18]	73.27	65	NR	NR	cefotaxime	74	Cefotaxime/metronidazole or ornidazole	72
Figueras-Felip 1984[19]	78.49	69.97	NR	49.46	Neomycin/erythromycin	45	Gentamicin/metronidazole	48
Gerner 1989[20]	71.24	61.45	10.76	50.64	Doxycycline/tinidazole	116	Doxycycline	107
Gortz 1990[21]	65%	NR	NR	NR	Moxalactam	60	Ciprofloxacin	60
Gorttrup 1985[22]	90.37	64.57	NR	NR	Placebo	41	Metronidazole	46
Gruner 1980[23]	77.89	NR	NR	51.58	Tinidazole/doxycycline	44	doxycycline	51
Hagen 1980[24]	73.68	67.86	NR	36.84	Metronidazole	17	Placebo	21
Hakansson 1993[25]	71.25	77	NR	43.74	cefotaxime	336	Cefotaxime/metronidazole	324
Hall 1989[26]	37.05	57.01	NR	29.25	Augmentin (amoxycillin+ clavulanate)	196	Gentamicin/metronidazole	204
Haverkorn 1985[27]	NR	NR	NR	NR	Metronidazole/netilmicin	12	Metronidazole/cefuroxime	13
Hershman 1990[28]	128	61	17	73.8	cefotetan	75	piperacillin	78
Hinchey 1987[29]	NR	67	NR	NR	moxalactam	64	Neomycin/metronidazole	67
Hjalmarsson 2015[30]	NR	70.15	1.33	52.79	trimethoprim-sulfamethoxazole/metronidazole (TSM)	533	Cefuroxime/metronidazole	540
Hoffmann 1981[31]	NR	68.5	NA	56.9	cefoxitin	32	placebo	33
Hojer 1978[32]	NR	NR	NR	NR	doxycycline	58	placebo	60
Hojer 1981[33]	69.77	61.49	NR	41.86	tinidazole	22	doxycycline	21

© 2026 Motaghi S et al. *JAMA Network Open*.

Hunt 1979[34]	85.91	NR	NR	30.98	Tinidazole	40	placebo	31
Itani 2006[35]	72.2	60.9	13.89	56.25	ertapenem	451	cefotetan	450
Iversson 1982[36]	0	58	NR	52.94	cefoxitin	55	doxycycline	47
Jagelman 1985[37]	42.5	54	34.8	55.2	Metronidazole	44	placebo	43
Jagelman 1982[38]	47.5	49	NA	44.4	Cefotaxime	34	Cefotaxime	33
Jagelman 1987[39]	NR	52.5	NA	69.8	Piperacillin sodium	43	Cefoxitin sodium	43
Jones 1987[40]	NR	NA	NA	NA	cefazolin	35	cefoxitin	36
Karran 1993[41]	78.2	67.6	11.5	50.43	imipenem	135	imipenem	138
Keighley 1983[42]	100	NR	NR	NR	Mezclocillin/metronidazole	40	Cefuroxime/metronidazole	33
Keighley 1976[43]	40.32	NR	NR	40.32	lincomycin	33	none	29
Kingston 1989[44]	62.88	65.4	13.5	49.34	latamoxef	121	Cefuroxime/metronidazole	108
Kling 1985[45]	53.78	64.2	12.9	51.56	metronidazole	29	Doxycycline	35
Kwok 1993[46]	93.9	60.6	13.5	50	amoxicillin/clavulanic acid	76	cefotaxime and metronidazole	88
Lauridsen 1988[47]	82	73.9	NR	37	Penicillin-Streptomycin	48	Cefotaxime	52
Leandoer 1976[48]	NR	NR	NR	52.63	Doxycycline	36	Pen/strep	21
Leng 2014[49]	92.67	59.25	11.75	57.07	ertapenem	299	ceftriaxone/metronidazole	300
Lindhagen 1984[50]	51.02	NA	NA	NA	metronidazole	23	Metronidazole/fosfomycin	26
Lozano 1985[51]	NR	60.27	13.31	46.66	Gentamycin+Lincomvcin	30	Gentamycin/Clindamycin	30
Luke 1991[52]	NR	57	NA	NA	Ceftriaxone	249	Ampicillin/metronidazole	247

© 2026 Motaghi S et al. *JAMA Network Open*.

Lumley 1992[53]	50,3	55	2	47	Ceftriaxone	94	Cefazolin	96
Matikainen 1993[54]	65.1	63	NA	42	Ceftriaxone/tinidatsole	315	netimycin/tinidatsole	313
McCulloch 1986[55]	67.44	59.96	NA	NA	Gentamicin/metronidazole	45	moxalactam	41
McDermott 1981[56]	87.5	59.76	15.24	54.17	Cephazoline/metronidazole	22	cephazolin	26
McLeish 1987[57]	NA	63.51	0.71	57.73	timentin	87	tinidazole	90
Mehigan 1981[58]	62.83	NA	MA	NA	Penicillin/gentamicin/clyndamici group	38	cefamandole	36
Mitchell 1983[59]	92.86	68.82	NA	51.43	metronidazole	34	Metronidazole/cefuroxime	36
Mittermayer 1984[60]	68.33	58.8	NA	58.33	Cefuroxime/metronidazole	27	metronidazole	33
Morris 1984[61]	NA	NA	NA	NA	latamoxef sodium	53	latamoxef sodium/metronidazole	56
Morris 1983[62]	61	61.96	1.41	47	Gentamicin/metronidazole	52	metronidazole	48
Morris 1990[63]	NA	NA	NA	NA	Aztreonam/metronidazole	71	Cefotaxime/metronidazole	70
Morris 1993[64]	NA	59.3	1.8	NR	Metronidazole/gentamicin	102	ceftriaxone	94
Morton 1989[65]	76	64	NA	49.34	cefotetan	278	Cefotetan/metronidazole	253
Mosimann 1997[66]	57.76	65.8	NA	53.11	amoxycillin/clavulanic acid	160	Gentamicin/clyndamicin	162
Mozzillo 1989[67]	86.56	62	13	56.61	Aztreonam/clindamycin	248	Gentamicin/clindamycin	247
Nyam 1995[68]	90.5	62.4	17.5	62.5	Amoxycillin/clavulanic acid group	100	Ceftriaxone/metronidazole	100
Offer 1988[69]	80.28	NR	NR	63.38	Ciprofloxacin/metronidazole	34	Cefazolin/metronidazole	36
Ofstad 1980[70]	NR	65	NR	43.67	Tinidazole	58	Tinidazole/doxycycline	70
Olsen 1983[71]	NR	65.3	12.45	51.11	Metronidazole	69	Placebo	66

© 2026 Motaghi S et al. *JAMA Network Open*.

Pacelli 1991[72]	100	66.8	12.42	62.29	imipenem-cilastatin	31	Cefuroxime/metronidazole	30
Panichi 1982[73]	62.16	NA	NA	NA	Cephalothin	28	Cefoxitin	23
Perrott 1985[74]	NR	NR	NR	NR	Metronidazole/Penicillin/obramycin	26	cefoxitin	27
Petrelli 1988[75]	100	59	NR	50	cefamandole	34	Placebo	36
Plouffe 1985[76]	NR	48	17	35%	cefazolin	24	moxalactam	26
Reers 1989[77]	NR	NR	MR	NR	piperacillin	89	latamoxef	80
Renner 1989[78]	NR	NR	35-93	49.95	ceftriaxone	52	Cephazolin/metronidazole	56
Rodolico 1991[79]	88.41	63.52	7.96	NR	Clindamycin/aztreonam	66	gentamicin	72
Roland 1985[80]	100	67	16	NR	Metronidazole	58	Metronidazole/Ampicillin	58
Rorbaek-Madsen 1988[81]	75.56	67.95	NR	51.13	cefoxotin	177	Ampicillin/Metronidazole	175
Rowe-Jones 1990[82]	66.15	68	18.52	48.56	Cefotaxime/metronidazole	471	Cefuroxime/metronidazole	472
Sato 2009[83]	100	63.5	9.96	56	cefotiam	51	Placebo	49
Shatney 1984[84]	NR	44.50	NR	36.25	Cefotaxime	79	Cefoxitin	81
Shimizu 2010[85]	NR	71.00	35-86	51.11	Cefmetazole	48	Flomoxef	43
Skipper 1992[86]	NR	NR	NR	49.04	Cefotetan	68	Cefuroxime/metronidazole	36
Slama 1979[87]	55.88	NR	NR	NR	Cefamandole	18	Cephalothin	16
Solhaug 1983[88]	NR	NR	NR	NR	Doxycycline	75	Metronidazole	72
Stubbs 1987[89]	67.27	72.71	NA	56.36	mezlocillin	54	Cefuroxime/metronidazole	56

© 2026 Motaghi S et al. *JAMA Network Open*.

Taylor 1994[90]	79	66.6	12.9	48	Ciprofloxacin	189	placebo	192
TNSG 1985[91]	87	65.6	range 14-80	51	Tinidazole/doxycycline	132	Tinidazole	135
Thomas 1985[92]	75	64	range 32-92	52	Latamoxef	60	Cephazolin/metronidazole	60
Tornqvist 1985[93]	NR	63.4	range 16-93	49	Cefuroxime	59	Cefuroxime/metronidazole	63
Ulrich 1981[94]	73	66.7	range 21-92	53	Clindamycin phosphate/kanamycin	25	Placebo	24
UoM 1986[95]	NR	65.5	NR	49	Ticarcillin	131	Tinidazole	130
UoM 1987[96]	NA	63.5	NR	58%	Timentin	101	Tinidazole	102
Utley 1984[97]	NR	49	NR	24	Cefoxitin	47	Placebo	47
Viddal 1980[98]	47.62	63.75	1.06	NR	Doxycycline/Tinidazole	21	Doxycycline	21
Walker 1988[99]	70.42	63.51	NR	48.36	piperacillin	108	Netilmicin/metronidazole	105
Watt-Boolsen 1979[100]	67.88	NR	NR	44.53	metronidazole	67	oxytetracycline	70
Weidema 1985[101]	NR	59.54	14.74	36.59	Gentamicin/metronidazole	21	metronidazole	20
Wetterfors 1980[102]	40	NR	NR	NR	doxycycline	58	placebo	60
Willis 1977[103]	78.26	NR	NR	56.52	metronidazole	27	placebo	19
Wohlfahrt 1987[104]	NR	63	NR	40	ceftriaxone	30	Cefotiam/gentamicin/metronidazole	30
Zanella 2000[105]	77.72	66.00	12.17	53.98	cefepime	308	ceftriaxone	308

*This symbol represents "and". [A] three-arm study, but merged to two because of the same node for two arms. [b] Three-arm study, but couldn't insert the third arm information because of space restriction

© 2026 Motaghi S et al. *JAMA Network Open*.

eTable 2. Node Definitions for Antibiotic Regimens in the Network Meta-Analysis

Node	Antibiotic class/Combination	Included Antibiotics
AMILIN	Aminoglycosides and Lincosamides	Gentamycin+Clindamycin<br>Gentamycin+Lincomycin<br>Kanamycin+Clindamycin
AMIMAC	Aminoglycosides and Macrolides	Erythromycin+Tobramycin<br>Erythromycin+Neomycin
AMINO	Aminoglycosides	Gentamycin
BRPEN	Broad-spectrum penicillins	Ampicillin+Sulbactam<br>Co-amoxiclav<br>Timentin<br>Mezlocillin
CARBA	Carbapenems	Ertapenem<br>Imipenem<br>Cefmetazole
FGCEPH	First-generation cephalosporin	Cephalothin<br>Cefazolin
FLUORQ	Fluoroquinolones	Ciprofloxacin
FORTGC	Fourth-generation cephalosporin	Cefepime
LINCO	Lincosamides	Lincomycin
LINMON	Lincosamides and Monobactams	Clindamicin+Aztreonam
MEASGC	Metronidazole, Aminoglycosides and Second-generation cephalosporins and	Cefotiam+Metronidazole+Gentamicin
METAMI	Metronidazole and Aminoglycosides	Metronidazole+Gentamycin<br>Metronidazole+Tobramycin<br>Metronidazole+Netilmicin<br>Metronidazole+Neomycin<br>Tinidatsole+Netilmycin
METBRO	Metronidazole and Broad-spectrum penicillins	Matronidazole+Ampicilin<br>Mezlocillin+Metronidazole
METFGC	Metronidazole and First-generation cephalosporins	Metronidazole+cefazolin
METFQ	Metronidazole and Fluoroquinolones	Metronidazole+Ciprofloxacin<br>Metronidazole+Levofloxacin
METMON	Metronidazole and Monobactams	Metronidazole+Aztreonam
METPAM	Metronidazole, Penicillin, and Aminoglycoside	Metronidazole+Penicillin+Tobramycin
METPEN	Metronidazole and Penicillin	Mezlocillin+Metronidazole
METPHO	Metronidazole and Phosphonic acid	Metronidazole+Fosfomycin
METRO	Metronidazole	Metronidazole<br>Tinidazole
METSGC	Metronidazole and Second-generation cephalosporins	Metronidazole+Cefuroxime<br>Tinidazole+Cephamandole<br>Metronidazole+Cefotetan
METSUL	Metronidazole and Sulfonamide	Trimethoprim-sulfamethoxazole +Metronidazole (TSM)
METTET	Tetracyclines and Metronidazole	Doxycycline+Metronidazole<br>Tinidazole+Doxycycline
METTGC	Metronidazole and Third-generation cephalosporins	Metronidazole+Ceftriaxone<br>Metronidazole+Cefotaxime<br>Tinidatsole+Ceftriaxone<br>Metronidazole+Cephazolin<br>Metronidazole+Latamoxef sodium

© 2026 Motaghi S et al. *JAMA Network Open*.

METNOB	Oxacephem	Flomoxef
PENALI	Penicillin, Aminoglycosides, and Lincosamide	Penicillin+Gentamicin+Clyndamicin
PENAMI	Penicillin and Aminoglycosides	Penicillin+Streptomycin
PENIC	Penicillin	Piperacillin
SGGEPH	Second-generation cephalosporins	Cefoxitin<br>Cefotetan<br>Cefamandole<br>Cefotiam<br>Cefuroxime
TETRA	Tetracyclines	Doxycycline<br>Oxytetracycline
TGCEPH	Third-generation cephalosporins	Latamoxef<br>Cefotaxime<br>Ceftriaxone<br>Moxalactam<br>Cephazolin
XPLACB	Placebo or no antibiotic	-

© 2026 Motaghi S et al. *JAMA Network Open*.

eTable 3. Risk of Bias Assessments for Included Trials

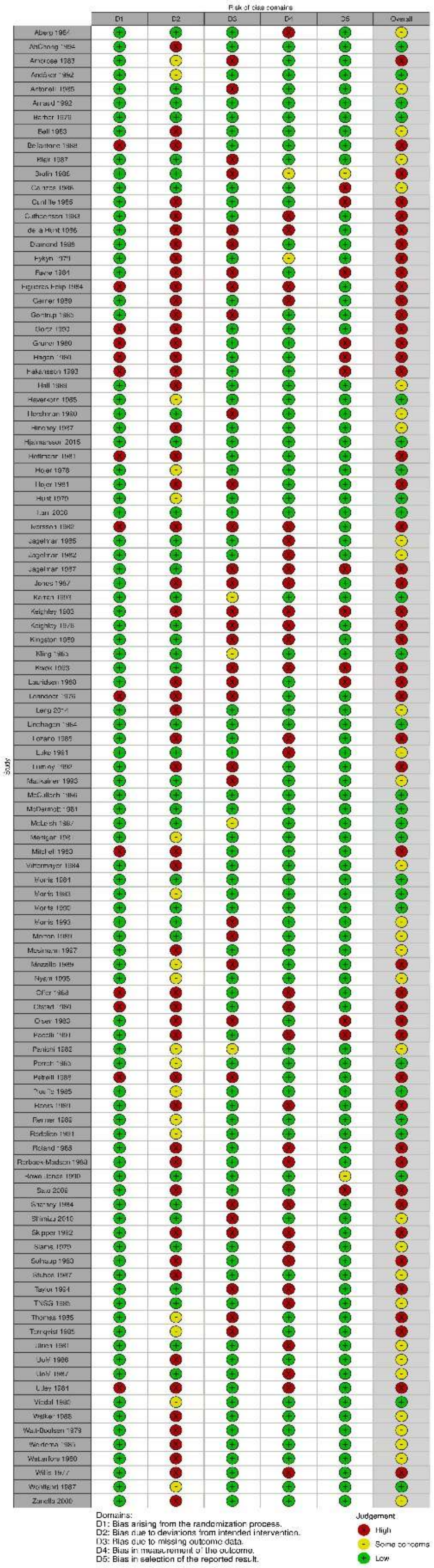

eTable 4. League Table for Surgical Site Infection—Relative Risk (95% CI)

**AMILIN**	1.15 (0.37,3.51)	0.73 (0.18,3.06)	1.03 (0.59,1.79)	0.94 (0.46,1.93)	1.06 (0.49,2.27)	2.44 (0.95,6.28)	1.07 (0.36,3.18)	1.27 (0.32,5.03)	0.47 (0.19,1.14)	2.13 (0.17,26.63)	1.66 (0.91,3.02)	1.03 (0.46,2.28)	1.15 (0.25,5.23)	0.81 (0.07,8.92)	2.81 (0.86,9.14)	2.37 (0.69,8.09)	1.81 (0.62,5.25)	0.29 (0.08,1.08)	1.82 (1.01,3.29)	1.06 (0.58,1.94)	2.06 (0.71,6.02)	0.78 (0.32,1.88)	1.11 (0.58,2.15)	0.79 (0.14,4.53)	1.17 (0.11,12.50)	3.02 (0.65,14.07)	1.59 (0.74,3.45)	1.37 (0.79,2.36)	1.28 (0.65,2.51)	1.07 (0.57,1.99)	3.97 (2.17,7.25)
0.87 (0.28,2.68)	**AMIMAC**	0.64 (0.11,3.89)	0.90 (0.32,2.51)	0.82 (0.27,2.52)	0.93 (0.29,2.91)	2.13 (0.59,7.75)	0.93 (0.24,3.69)	1.11 (0.21,5.72)	0.41 (0.10,1.69)	1.86 (0.13,26.58)	1.45 (0.56,3.72)	0.90 (0.28,2.89)	1.00 (0.18,5.64)	0.71 (0.06,8.95)	2.45 (0.58,10.37)	2.07 (0.46,9.38)	1.58 (0.40,6.20)	0.25 (0.05,1.21)	1.59 (0.56,4.48)	0.93 (0.33,2.65)	1.80 (0.46,7.10)	0.68 (0.20,2.32)	0.97 (0.34,2.81)	0.69 (0.10,4.83)	1.02 (0.08,12.82)	2.64 (0.45,15.31)	1.39 (0.45,4.31)	1.20 (0.42,3.37)	1.12 (0.38,3.33)	0.93 (0.33,2.64)	3.46 (1.18,10.19)
1.36 (0.33,5.67)	1.56 (0.26,9.48)	**AMINO**	1.40 (0.31,6.40)	1.28 (0.26,6.24)	1.44 (0.29,7.19)	3.32 (0.61,18.20)	1.46 (0.24,8.69)	1.73 (0.24,12.44)	0.64 (0.21,1.93)	2.91 (0.16,52.44)	2.26 (0.49,10.49)	1.40 (0.28,7.08)	1.56 (0.20,12.43)	1.10 (0.07,17.86)	3.82 (0.61,24.10)	3.23 (0.50,20.96)	2.46 (0.42,14.45)	0.39 (0.06,2.71)	2.48 (0.54,11.47)	1.45 (0.31,6.73)	2.81 (0.48,16.56)	1.06 (0.20,5.61)	1.52 (0.32,7.22)	1.07 (0.11,10.15)	1.59 (0.10,25.13)	4.11 (0.51,33.23)	2.17 (0.43,10.85)	1.87 (0.41,8.47)	1.74 (0.36,8.35)	1.45 (0.31,6.81)	5.40 (1.16,25.13)
0.97 (0.56,1.69)	1.11 (0.40,3.11)	0.71 (0.16,3.26)	**BRPEN**	0.91 (0.51,1.63)	1.03 (0.54,1.96)	2.37 (0.99,5.66)	1.04 (0.38,2.84)	1.23 (0.33,4.67)	0.45 (0.16,1.28)	2.07 (0.17,24.95)	1.61 (1.07,2.41)	1.00 (0.51,1.96)	1.11 (0.26,4.77)	0.79 (0.07,8.33)	2.73 (0.92,8.09)	2.30 (0.72,7.37)	1.76 (0.67,4.63)	0.28 (0.08,0.98)	1.77 (1.17,2.66)	1.03 (0.69,1.55)	2.01 (0.76,5.32)	0.76 (0.35,1.64)	1.08 (0.67,1.74)	0.76 (0.14,4.17)	1.14 (0.11,11.79)	2.94 (0.67,12.92)	1.55 (0.81,2.97)	1.33 (0.91,1.94)	1.24 (0.73,2.13)	1.04 (0.66,1.63)	3.86 (2.36,6.30)
1.07 (0.52,2.20)	1.22 (0.40,3.76)	0.78 (0.16,3.82)	1.10 (0.61,1.97)	**CARBA**	1.13 (0.54,2.38)	2.60 (1.00,6.78)	1.14 (0.39,3.34)	1.35 (0.34,5.45)	0.50 (0.16,1.55)	2.28 (0.18,28.18)	1.77 (0.96,3.26)	1.10 (0.51,2.37)	1.22 (0.27,5.50)	0.86 (0.08,9.43)	2.99 (0.95,9.44)	2.53 (0.75,8.51)	1.93 (0.70,5.33)	0.31 (0.08,1.15)	1.94 (1.07,3.51)	1.14 (0.68,1.89)	2.20 (0.79,6.12)	0.83 (0.34,2.01)	1.19 (0.65,2.17)	0.84 (0.17,4.13)	1.25 (0.12,13.32)	3.22 (0.69,14.99)	1.70 (0.79,3.63)	1.46 (0.87,2.44)	1.37 (0.69,2.69)	1.14 (0.63,2.06)	4.23 (2.24,8.00)
0.94 (0.44,2.03)	1.08 (0.34,3.41)	0.69 (0.14,3.45)	0.97 (0.51,1.85)	0.89 (0.42,1.86)	**FGCEPH**	2.30 (0.88,6.05)	1.01 (0.36,2.86)	1.20 (0.29,4.90)	0.44 (0.14,1.41)	2.01 (0.16,24.62)	1.56 (0.82,2.99)	0.97 (0.43,2.19)	1.08 (0.24,4.78)	0.76 (0.07,8.25)	2.65 (0.82,8.58)	2.24 (0.64,7.78)	1.71 (0.58,5.03)	0.27 (0.07,1.05)	1.72 (0.90,3.28)	1.00 (0.54,1.88)	1.95 (0.66,5.77)	0.74 (0.29,1.84)	1.05 (0.55,2.01)	0.74 (0.13,4.31)	1.10 (0.10,11.95)	2.85 (0.61,13.43)	1.50 (0.69,3.30)	1.29 (0.72,2.33)	1.21 (0.59,2.49)	1.01 (0.59,1.72)	3.74 (1.91,7.35)
0.41 (0.16,1.05)	0.47 (0.13,1.71)	0.30 (0.05,1.65)	0.42 (0.18,1.01)	0.38 (0.15,1.00)	0.43 (0.17,1.14)	**FLUORQ**	0.44 (0.13,1.50)	0.52 (0.12,2.23)	0.19 (0.05,0.69)	0.87 (0.07,11.64)	0.68 (0.28,1.63)	0.42 (0.15,1.15)	0.47 (0.09,2.38)	0.33 (0.03,3.92)	1.15 (0.30,4.35)	0.97 (0.24,3.88)	0.74 (0.22,2.54)	0.12 (0.03,0.50)	0.75 (0.33,1.71)	0.44 (0.18,1.03)	0.85 (0.25,2.91)	0.32 (0.11,0.91)	0.46 (0.19,1.12)	0.32 (0.05,2.07)	0.48 (0.04,5.51)	1.24 (0.24,6.40)	0.65 (0.24,1.76)	0.56 (0.24,1.30)	0.52 (0.22,1.27)	0.44 (0.19,1.02)	1.63 (0.75,3.51)
0.93 (0.31,2.78)	1.07 (0.27,4.22)	0.69 (0.12,4.08)	0.96 (0.35,2.62)	0.88 (0.30,2.56)	0.99 (0.35,2.80)	2.28 (0.67,7.81)	**FORTGC**	1.18 (0.24,5.94)	0.44 (0.11,1.76)	1.99 (0.15,26.94)	1.55 (0.57,4.18)	0.96 (0.31,2.93)	1.07 (0.21,5.56)	0.76 (0.06,9.06)	2.62 (0.65,10.52)	2.21 (0.51,9.69)	1.69 (0.45,6.35)	0.27 (0.06,1.27)	1.70 (0.62,4.65)	0.99 (0.37,2.67)	1.93 (0.51,7.28)	0.73 (0.22,2.41)	1.04 (0.39,2.79)	0.73 (0.11,5.02)	1.09 (0.09,13.43)	2.82 (0.50,15.87)	1.49 (0.50,4.46)	1.28 (0.48,3.42)	1.20 (0.42,3.45)	1.00 (0.41,2.44)	3.71 (1.32,10.39)
0.79 (0.20,3.13)	0.90 (0.17,4.67)	0.58 (0.08,4.17)	0.81 (0.21,3.08)	0.74 (0.18,2.98)	0.84 (0.20,3.42)	1.92 (0.45,8.28)	0.84 (0.17,4.23)	**LINCO**	0.37 (0.07,1.89)	1.68 (0.10,27.38)	1.31 (0.34,4.99)	0.81 (0.20,3.36)	0.90 (0.13,6.22)	0.64 (0.04,9.30)	2.21 (0.41,11.87)	1.87 (0.34,10.37)	1.43 (0.29,7.03)	0.23 (0.04,1.31)	1.43 (0.39,5.24)	0.84 (0.22,3.18)	1.63 (0.33,8.05)	0.61 (0.14,2.61)	0.88 (0.22,3.44)	0.62 (0.07,5.15)	0.92 (0.07,12.89)	2.38 (0.35,16.33)	1.26 (0.30,5.21)	1.08 (0.29,4.02)	1.01 (0.27,3.83)	0.84 (0.22,3.22)	3.13 (0.91,10.80)
2.14 (0.87,5.25)	2.45 (0.59,10.17)	1.57 (0.52,4.77)	2.20 (0.78,6.22)	2.01 (0.65,6.24)	2.27 (0.71,7.23)	5.22 (1.44,18.94)	2.29 (0.57,9.27)	2.71 (0.53,13.89)	**LINMON**	4.57 (0.32,66.04)	3.55 (1.23,10.26)	2.20 (0.67,7.17)	2.45 (0.43,14.15)	1.73 (0.13,22.29)	6.01 (1.38,26.11)	5.07 (1.13,22.86)	3.87 (0.97,15.36)	0.62 (0.13,3.00)	3.89 (1.35,11.20)	2.28 (0.79,6.58)	4.42 (1.11,17.61)	1.67 (0.48,5.77)	2.38 (0.80,7.13)	1.68 (0.24,11.90)	2.51 (0.20,31.28)	6.47 (1.10,37.95)	3.41 (1.06,10.94)	2.93 (1.05,8.20)	2.74 (0.91,8.27)	2.28 (0.78,6.69)	8.49 (2.93,24.59)
0.47 (0.04,5.85)	0.54 (0.04,7.66)	0.34 (0.02,6.20)	0.48 (0.04,5.80)	0.44 (0.04,5.44)	0.50 (0.04,6.07)	1.14 (0.09,15.21)	0.50 (0.04,6.78)	0.59 (0.04,9.66)	0.22 (0.02,3.16)	**MEASGC**	0.78 (0.06,9.31)	0.48 (0.04,6.08)	0.54 (0.03,8.92)	0.38 (0.01,11.02)	1.32 (0.09,18.94)	1.11 (0.07,16.74)	0.85 (0.06,11.79)	0.14 (0.01,2.12)	0.85 (0.07,10.27)	0.50 (0.04,5.97)	0.97 (0.07,13.49)	0.37 (0.03,4.78)	0.52 (0.04,6.24)	0.37 (0.02,7.24)	0.55 (0.02,16.23)	1.42 (0.08,24.65)	0.75 (0.06,9.34)	0.64 (0.05,7.66)	0.60 (0.05,7.39)	0.50 (0.04,5.77)	1.86 (0.15,22.62)
0.60 (0.33,1.10)	0.69 (0.27,1.78)	0.44 (0.10,2.06)	**0.62 (0.41,0.93)**	0.57 (0.31,1.05)	0.64 (0.33,1.22)	1.47 (0.61,3.53)	0.65 (0.24,1.74)	0.76 (0.20,2.92)	**0.28 (0.10,0.81)**	1.29 (0.11,15.43)	**METAMI**	0.62 (0.31,1.24)	0.69 (0.16,2.95)	0.49 (0.05,5.16)	1.69 (0.57,5.04)	1.43 (0.44,4.65)	1.09 (0.41,2.93)	0.17 (0.05,0.61)	1.10 (0.72,1.68)	0.64 (0.41,1.01)	1.25 (0.46,3.37)	0.47 (0.22,1.02)	0.67 (0.42,1.09)	0.47 (0.09,2.62)	0.71 (0.07,7.37)	1.82 (0.41,8.02)	0.96 (0.52,1.79)	0.83 (0.54,1.27)	0.77 (0.45,1.32)	0.64 (0.42,0.99)	2.39 (1.44,3.99)
0.97 (0.44,2.16)	1.11 (0.35,3.59)	0.71 (0.14,3.61)	1.00 (0.51,1.97)	0.91 (0.42,1.97)	1.03 (0.46,2.32)	2.37 (0.87,6.47)	1.04 (0.34,3.18)	1.23 (0.30,5.12)	0.45 (0.14,1.48)	2.08 (0.16,26.21)	1.61 (0.81,3.22)	**METBRO**	1.11 (0.24,5.18)	0.79 (0.07,8.77)	2.73 (0.81,9.18)	2.31 (0.65,8.16)	1.76 (0.59,5.24)	0.28 (0.07,1.08)	1.77 (0.92,3.39)	1.04 (0.54,1.97)	2.01 (0.67,6.01)	0.76 (0.30,1.91)	1.08 (0.53,2.21)	0.76 (0.13,4.49)	1.14 (0.10,12.44)	2.94 (0.61,14.06)	1.55 (0.68,3.56)	1.33 (0.72,2.48)	1.25 (0.60,2.60)	1.04 (0.53,2.03)	3.86 (1.92,7.78)
0.87 (0.19,3.99)	1.00 (0.18,5.64)	0.64 (0.08,5.10)	0.90 (0.21,3.85)	0.82 (0.18,3.69)	0.92 (0.21,4.08)	2.13 (0.42,10.81)	0.93 (0.18,4.85)	1.11 (0.16,7.62)	0.41 (0.07,2.35)	1.86 (0.11,30.94)	1.45 (0.34,6.17)	0.90 (0.19,4.17)	**METFGC**	0.71 (0.11,4.53)	2.45 (0.43,14.03)	2.07 (0.34,12.69)	1.58 (0.29,8.58)	0.25 (0.04,1.64)	1.59 (0.37,6.83)	0.93 (0.22,3.95)	1.80 (0.33,9.83)	0.68 (0.14,3.37)	0.97 (0.23,4.12)	0.69 (0.08,6.14)	1.02 (0.07,15.54)	2.64 (0.35,19.99)	1.39 (0.30,6.37)	1.20 (0.28,5.06)	1.12 (0.25,4.99)	0.93 (0.23,3.72)	3.46 (0.79,15.20)
1.24 (0.11,13.64)	1.42 (0.11,17.95)	0.91 (0.06,14.71)	1.27 (0.12,13.49)	1.16 (0.11,12.68)	1.31 (0.12,14.15)	3.02 (0.26,35.63)	1.32 (0.11,15.87)	1.57 (0.11,22.85)	0.58 (0.04,7.43)	2.64 (0.09,76.69)	2.05 (0.19,21.65)	1.27 (0.11,14.17)	1.42 (0.22,9.09)	**METFQ**	3.47 (0.27,44.43)	2.93 (0.22,39.36)	2.23 (0.18,27.62)	0.36 (0.03,5.00)	2.25 (0.21,23.90)	1.32 (0.12,13.88)	2.55 (0.21,31.62)	0.96 (0.08,11.19)	1.38 (0.13,14.50)	0.97 (0.05,17.21)	1.45 (0.05,39.10)	3.73 (0.24,58.39)	1.97 (0.18,21.77)	1.69 (0.16,17.82)	1.58 (0.15,17.22)	1.32 (0.13,13.40)	4.91 (0.46,52.77)
0.36 (0.11,1.16)	0.41 (0.10,1.73)	0.26 (0.04,1.65)	0.37 (0.12,1.09)	0.33 (0.11,1.05)	0.38 (0.12,1.22)	0.87 (0.23,3.29)	0.38 (0.10,1.53)	0.45 (0.08,2.42)	**0.17 (0.04,0.72)**	0.76 (0.05,10.95)	0.59 (0.20,1.76)	0.37 (0.11,1.23)	0.41 (0.07,2.34)	0.29 (0.02,3.69)	**METMON**	0.84 (0.18,3.96)	0.64 (0.16,2.59)	0.10 (0.02,0.52)	0.65 (0.21,1.95)	0.38 (0.13,1.12)	0.74 (0.18,2.97)	0.28 (0.08,1.00)	0.40 (0.15,1.06)	0.28 (0.04,2.00)	0.42 (0.03,5.35)	1.08 (0.18,6.47)	0.57 (0.17,1.88)	0.49 (0.16,1.45)	0.46 (0.14,1.45)	0.38 (0.13,1.10)	1.41 (0.46,4.39)
0.42 (0.12,1.44)	0.48 (0.11,2.19)	0.31 (0.05,2.01)	0.43 (0.14,1.39)	0.40 (0.12,1.33)	0.45 (0.13,1.55)	1.03 (0.26,4.11)	0.45 (0.10,1.98)	0.53 (0.10,2.97)	**0.20 (0.04,0.89)**	0.90 (0.06,13.57)	0.70 (0.21,2.28)	0.43 (0.12,1.53)	0.48 (0.08,2.97)	0.34 (0.03,4.58)	1.18 (0.25,5.55)	**METPAM**	0.76 (0.18,3.26)	0.12 (0.02,0.64)	0.77 (0.24,2.47)	0.45 (0.14,1.43)	0.87 (0.20,3.74)	0.33 (0.09,1.25)	0.47 (0.14,1.55)	0.33 (0.04,2.46)	0.49 (0.04,6.40)	1.27 (0.20,8.00)	0.67 (0.19,2.34)	0.58 (0.19,1.73)	0.54 (0.16,1.81)	0.45 (0.14,1.46)	1.67 (0.51,5.46)
0.55 (0.19,1.61)	0.63 (0.16,2.49)	0.41 (0.07,2.39)	0.57 (0.22,1.50)	0.52 (0.19,1.44)	0.59 (0.20,1.73)	1.35 (0.39,4.63)	0.59 (0.16,2.23)	0.70 (0.14,3.46)	0.26 (0.07,1.03)	1.18 (0.08,16.45)	0.92 (0.34,2.47)	0.57 (0.19,1.69)	0.63 (0.12,3.45)	0.45 (0.04,5.54)	1.55 (0.39,6.26)	1.31 (0.31,5.62)	**METPEN**	0.16 (0.03,0.73)	1.01 (0.38,2.65)	0.59 (0.24,1.42)	1.14 (0.33,3.99)	0.43 (0.13,1.39)	0.62 (0.23,1.66)	0.43 (0.07,2.88)	0.65 (0.05,7.87)	1.67 (0.30,9.35)	0.88 (0.29,2.64)	0.76 (0.29,1.96)	0.71 (0.25,1.98)	0.59 (0.22,1.57)	2.20 (0.80,6.00)
3.47 (0.93,12.98)	3.97 (0.83,19.11)	2.55 (0.37,17.61)	3.57 (1.02,12.46)	3.25 (0.87,12.17)	3.68 (0.96,14.12)	8.47 (2.00,35.79)	3.71 (0.79,17.51)	4.40 (0.76,25.38)	1.62 (0.33,7.89)	7.41 (0.47,116.33)	**5.75 (1.64,20.15)**	3.57 (0.93,13.72)	3.98 (0.61,25.96)	2.81 (0.20,39.38)	**9.74 (1.94,48.98)**	**8.23 (1.57,43.19)**	**6.27 (1.36,28.89)**	**METPHO**	6.31 (1.92,20.76)	3.69 (1.06,12.87)	7.17 (1.55,33.10)	2.71 (0.96,7.66)	3.87 (1.07,13.97)	2.72 (0.34,21.60)	4.06 (0.30,55.44)	10.48 (1.66,66.03)	5.53 (1.43,21.33)	4.75 (1.37,16.45)	4.44 (1.34,14.69)	3.70 (1.04,13.14)	13.77 (3.99,47.53)
**0.55 (0.30,0.99)**	0.63 (0.22,1.78)	0.40 (0.09,1.87)	**0.57 (0.38,0.85)**	**0.52 (0.29,0.93)**	0.58 (0.30,1.11)	1.34 (0.59,3.07)	0.59 (0.22,1.61)	0.70 (0.19,2.55)	**0.26 (0.09,0.74)**	1.17 (0.10,14.14)	0.91 (0.59,1.40)	0.57 (0.30,1.08)	0.63 (0.15,2.71)	0.44 (0.04,4.73)	1.54 (0.51,4.66)	1.30 (0.41,4.19)	0.99 (0.38,2.62)	**0.16 (0.05,0.52)**	**METRO**	0.59 (0.39,0.88)	1.14 (0.43,3.01)	0.43 (0.22,0.84)	0.61 (0.37,1.02)	0.43 (0.08,2.37)	0.64 (0.06,6.64)	1.66 (0.39,7.07)	0.88 (0.45,1.70)	0.75 (0.51,1.12)	0.70 (0.47,1.06)	0.59 (0.37,0.93)	2.18 (1.49,3.19)
0.94 (0.51,1.71)	1.08 (0.38,3.06)	0.69 (0.15,3.20)	0.97 (0.64,1.45)	0.88 (0.53,1.47)	1.00 (0.53,1.86)	2.29 (0.97,5.43)	1.01 (0.37,2.70)	1.19 (0.31,4.50)	0.44 (0.15,1.27)	2.00 (0.17,23.98)	1.56 (0.99,2.44)	0.97 (0.51,1.84)	1.08 (0.25,4.57)	0.76 (0.07,8.01)	2.64 (0.90,7.76)	2.23 (0.70,7.08)	1.70 (0.70,4.10)	**0.27 (0.08,0.94)**	**1.71 (1.14,2.55)**	**METSGC**	1.94 (0.80,4.70)	0.73 (0.34,1.58)	1.05 (0.66,1.65)	0.74 (0.14,3.94)	1.10 (0.11,11.37)	2.84 (0.65,12.45)	1.50 (0.78,2.87)	1.29 (0.90,1.85)	1.20 (0.71,2.05)	1.00 (0.66,1.53)	3.73 (2.30,6.05)
0.48 (0.17,1.41)	0.55 (0.14,2.19)	0.36 (0.06,2.09)	0.50 (0.19,1.32)	0.45 (0.16,1.26)	0.51 (0.17,1.52)	1.18 (0.34,4.07)	0.52 (0.14,1.96)	0.61 (0.12,3.04)	**0.23 (0.06,0.90)**	1.03 (0.07,14.41)	0.80 (0.30,2.17)	0.50 (0.17,1.49)	0.55 (0.10,3.03)	0.39 (0.03,4.85)	1.36 (0.34,5.49)	1.15 (0.27,4.93)	0.88 (0.25,3.05)	**0.14 (0.03,0.64)**	0.88 (0.33,2.33)	0.52 (0.21,1.25)	**METSUL**	0.38 (0.12,1.22)	0.54 (0.20,1.46)	0.38 (0.06,2.53)	0.57 (0.05,6.90)	1.46 (0.26,8.20)	0.77 (0.26,2.32)	0.66 (0.25,1.73)	0.62 (0.22,1.74)	0.52 (0.19,1.38)	1.92 (0.70,5.27)
1.28 (0.53,3.09)	1.47 (0.43,5.01)	0.94 (0.18,4.97)	1.32 (0.61,2.86)	1.20 (0.50,2.90)	1.36 (0.54,3.40)	3.13 (1.09,8.95)	1.37 (0.41,4.55)	1.63 (0.38,6.91)	0.60 (0.17,2.07)	2.74 (0.21,35.85)	2.13 (0.98,4.63)	1.32 (0.52,3.32)	1.47 (0.30,7.27)	1.04 (0.09,12.05)	**3.60 (1.00,12.98)**	3.04 (0.80,11.57)	2.32 (0.72,7.47)	0.37 (0.13,1.05)	**2.33 (1.19,4.57)**	1.37 (0.63,2.95)	2.65 (0.82,8.57)	**METTET**	1.43 (0.62,3.27)	1.01 (0.16,6.23)	1.50 (0.13,16.90)	3.87 (0.82,18.25)	2.04 (0.81,5.16)	1.76 (0.82,3.76)	1.64 (0.85,3.19)	1.37 (0.62,3.04)	5.09 (2.41,10.75)
0.90 (0.46,1.73)	1.03 (0.36,2.97)	0.66 (0.14,3.13)	0.92 (0.58,1.48)	0.84 (0.46,1.54)	0.95 (0.50,1.82)	2.19 (0.89,5.38)	0.96 (0.36,2.58)	1.14 (0.29,4.45)	0.42 (0.14,1.25)	1.92 (0.16,22.89)	1.49 (0.92,2.41)	0.92 (0.45,1.89)	1.03 (0.24,4.36)	0.73 (0.07,7.65)	2.52 (0.95,6.71)	2.13 (0.64,7.03)	1.62 (0.60,4.37)	**0.26 (0.07,0.93)**	1.63 (0.98,2.72)	0.96 (0.61,1.50)	1.85 (0.69,5.02)	0.70 (0.31,1.60)	**METTGC**	0.70 (0.13,3.88)	1.05 (0.10,11.08)	2.71 (0.60,12.19)	1.43 (0.72,2.85)	1.23 (0.77,1.97)	1.15 (0.62,2.12)	0.96 (0.63,1.45)	3.56 (2.01,6.30)
1.27 (0.22,7.34)	1.46 (0.21,10.28)	0.93 (0.10,8.87)	1.31 (0.24,7.16)	1.19 (0.24,5.89)	1.35 (0.23,7.85)	3.11 (0.48,19.98)	1.36 (0.20,9.32)	1.61 (0.19,13.43)	0.59 (0.08,4.21)	2.72 (0.14,53.49)	2.11 (0.38,11.66)	1.31 (0.22,7.70)	1.46 (0.16,13.08)	1.03 (0.06,18.26)	3.57 (0.50,25.54)	3.02 (0.41,22.42)	2.30 (0.35,15.27)	0.37 (0.05,2.91)	2.32 (0.42,12.70)	1.36 (0.25,7.24)	2.63 (0.40,17.49)	0.99 (0.16,6.14)	1.42 (0.26,7.81)	**MONOB**	1.49 (0.09,25.90)	3.85 (0.42,35.28)	2.03 (0.35,11.88)	1.74 (0.33,9.33)	1.63 (0.29,9.24)	1.36 (0.25,7.46)	5.05 (0.91,28.17)
0.85 (0.08,9.13)	0.98 (0.08,12.28)	0.63 (0.04,9.88)	0.88 (0.08,9.11)	0.80 (0.08,8.55)	0.91 (0.08,9.79)	2.08 (0.18,23.94)	0.91 (0.07,11.23)	1.08 (0.08,15.12)	0.40 (0.03,4.98)	1.82 (0.06,53.94)	1.42 (0.14,14.78)	0.88 (0.08,9.59)	0.98 (0.06,14.89)	0.69 (0.03,18.67)	2.40 (0.19,30.74)	2.03 (0.16,26.26)	1.54 (0.13,18.75)	0.25 (0.02,3.36)	1.55 (0.15,16.02)	0.91 (0.09,9.41)	1.76 (0.14,21.46)	0.67 (0.06,7.50)	0.95 (0.09,10.03)	0.67 (0.04,11.66)	**PENALI**	2.58 (0.17,39.59)	1.36 (0.13,14.78)	1.17 (0.12,11.84)	1.09 (0.10,11.52)	0.91 (0.09,9.50)	3.39 (0.33,34.72)
0.33 (0.07,1.54)	0.38 (0.07,2.20)	0.24 (0.03,1.96)	0.34 (0.08,1.50)	0.31 (0.07,1.45)	0.35 (0.07,1.65)	0.81 (0.16,4.17)	0.35 (0.06,1.99)	0.42 (0.06,2.88)	**0.15 (0.03,0.91)**	0.71 (0.04,12.31)	0.55 (0.12,2.42)	0.34 (0.07,1.63)	0.38 (0.05,2.88)	0.27 (0.02,4.19)	0.93 (0.15,5.59)	0.78 (0.13,4.93)	0.60 (0.11,3.35)	**0.10 (0.02,0.60)**	0.60 (0.14,2.56)	0.35 (0.08,1.55)	0.68 (0.12,3.83)	0.26 (0.05,1.22)	0.37 (0.08,1.66)	0.26 (0.03,2.38)	0.39 (0.03,5.95)	**PENAMI**	0.53 (0.11,2.51)	0.45 (0.10,1.97)	0.42 (0.10,1.73)	0.35 (0.08,1.55)	1.31 (0.30,5.73)
0.63 (0.29,1.36)	0.72 (0.23,2.23)	0.46 (0.09,2.30)	0.65 (0.34,1.24)	0.59 (0.28,1.26)	0.67 (0.30,1.46)	1.53 (0.57,4.14)	0.67 (0.22,2.01)	0.80 (0.19,3.30)	**0.29 (0.09,0.94)**	1.34 (0.11,16.77)	1.04 (0.56,1.94)	0.65 (0.28,1.48)	0.72 (0.16,3.30)	0.51 (0.05,5.62)	1.76 (0.53,5.84)	1.49 (0.43,5.19)	1.13 (0.38,3.39)	**0.18 (0.05,0.70)**	1.14 (0.59,2.21)	0.67 (0.35,1.28)	1.30 (0.43,3.89)	0.49 (0.19,1.24)	0.70 (0.35,1.40)	0.49 (0.08,2.89)	0.74 (0.07,7.99)	1.90 (0.40,9.04)	**PENIC**	0.86 (0.47,1.56)	0.80 (0.39,1.68)	0.67 (0.35,1.27)	2.49 (1.24,5.01)
0.73 (0.42,1.26)	0.84 (0.30,2.36)	0.54 (0.12,2.43)	0.75 (0.52,1.10)	0.68 (0.41,1.15)	0.77 (0.43,1.39)	1.78 (0.77,4.14)	0.78 (0.29,2.09)	0.93 (0.25,3.44)	**0.34 (0.12,0.95)**	1.56 (0.13,18.61)	1.21 (0.79,1.86)	0.75 (0.40,1.40)	0.84 (0.20,3.54)	0.59 (0.06,6.21)	2.05 (0.69,6.07)	1.73 (0.58,5.20)	1.32 (0.51,3.42)	**0.21 (0.06,0.73)**	1.33 (0.89,1.97)	0.78 (0.54,1.12)	1.51 (0.58,3.92)	0.57 (0.27,1.22)	0.81 (0.51,1.30)	0.57 (0.11,3.07)	0.85 (0.08,8.65)	2.21 (0.51,9.60)	1.16 (0.64,2.11)	**SGCEPH**	0.94 (0.56,1.55)	0.78 (0.52,1.18)	2.90 (1.87,4.48)
0.78 (0.40,1.53)	0.89 (0.30,2.66)	0.57 (0.12,2.74)	0.80 (0.47,1.37)	0.73 (0.37,1.44)	0.83 (0.40,1.70)	1.91 (0.79,4.62)	0.84 (0.29,2.41)	0.99 (0.26,3.75)	0.36 (0.12,1.10)	1.67 (0.14,20.51)	1.29 (0.75,2.22)	0.80 (0.38,1.68)	0.89 (0.20,4.00)	0.63 (0.06,6.87)	2.19 (0.69,6.95)	1.85 (0.55,6.21)	1.41 (0.50,3.95)	**0.23 (0.07,0.74)**	1.42 (0.95,2.13)	0.83 (0.49,1.41)	1.61 (0.57,4.53)	0.61 (0.31,1.18)	0.87 (0.47,1.60)	0.61 (0.11,3.47)	0.91 (0.09,9.62)	2.36 (0.58,9.65)	1.24 (0.60,2.59)	1.07 (0.64,1.77)	**TETRA**	0.83 (0.47,1.47)	3.10 (1.90,5.06)
0.94 (0.50,1.75)	1.07 (0.38,3.04)	0.69 (0.15,3.22)	0.96 (0.61,1.52)	0.88 (0.49,1.59)	0.99 (0.58,1.69)	2.29 (0.98,5.33)	1.00 (0.41,2.45)	1.19 (0.31,4.54)	0.44 (0.15,1.28)	2.00 (0.17,23.07)	**1.55 (1.01,2.40)**	0.96 (0.49,1.88)	1.07 (0.27,4.29)	0.76 (0.07,7.70)	2.63 (0.91,7.63)	2.22 (0.69,7.19)	1.69 (0.64,4.50)	**0.27 (0.08,0.96)**	**1.70 (1.07,2.71)**	1.00 (0.65,1.52)	1.94 (0.73,5.17)	0.73 (0.33,1.62)	1.04 (0.69,1.58)	0.74 (0.13,4.04)	1.10 (0.11,11.43)	2.83 (0.65,12.41)	1.49 (0.79,2.82)	1.28 (0.85,1.94)	1.20 (0.68,2.11)	**TGCEPH**	3.72 (2.22,6.21)
**0.25 (0.14,0.46)**	**0.29 (0.10,0.85)**	**0.19 (0.04,0.86)**	**0.26 (0.16,0.42)**	**0.24 (0.12,0.45)**	**0.27 (0.14,0.52)**	0.62 (0.28,1.33)	**0.27 (0.10,0.76)**	0.32 (0.09,1.10)	**0.12 (0.04,0.34)**	0.54 (0.04,6.55)	**0.42 (0.25,0.70)**	**0.26 (0.13,0.52)**	0.29 (0.07,1.27)	0.20 (0.02,2.19)	0.71 (0.23,2.20)	0.60 (0.18,1.95)	0.46 (0.17,1.24)	**0.07 (0.02,0.25)**	**0.46 (0.31,0.67)**	**0.27 (0.17,0.44)**	0.52 (0.19,1.43)	**0.20 (0.09,0.42)**	**0.28 (0.16,0.50)**	0.20 (0.04,1.10)	0.30 (0.03,3.02)	0.76 (0.17,3.32)	**0.40 (0.20,0.81)**	**0.35 (0.22,0.53)**	**0.32 (0.20,0.53)**	**0.27 (0.16,0.45)**	**XPLACB**

Node abbreviation: AMILIN: Aminoglycosides and Lincosamides, **AMIMAC**: Aminoglycosides and Macrolides, **AMINO**: Aminoglycosides, **BRPEN**: Broad-spectrum penicillins, **CARBA**: Carbapenems, **FGCEPH**: First-generation cephalosporin, **FLUORQ**: Fluoroquinolones, **FORTGC**: Fourth-generation cephalosporin, **LINCO**: Lincosamides, **LINMON**: Lincosamides and Monobactams, **MEASGC**: Metronidazole and Second-generation cephalosporins and Aminoglycosides, **METAMI**: Aminoglycosides and Metronidazole, **METBRO**: Metronidazole and Broad-spectrum penicillins, **METFGC**: Metronidazole and First-generation cephalosporins, **METFQ**: Metronidazole and Fluoroquinolones, **METMON**: Metronidazole and Monobactams, **METPAM**: Metronidazole, Penicillin, and Aminoglycoside, **METPEN**: Metronidazole and Penicillin, **METPHO**: Metronidazole and Phosphonic acid, **METRO**: Metronidazole, **METSGC**: Metronidazole and Second-generation cephalosporins, **METSUL**: Metronidazole and Sulfonamide, **METTET**: Tetracyclines and Metronidazole, **METTGC**: Metronidazole and Third-generation cephalosporins, **METNOB**: Oxacephem, **PENALI**: Penicillin, Aminoglycosides, and Lincosamide, **PENAMI**: Penicillin and Aminoglycosides, **PENIC**: Penicillin, **SGGEPH**: Second-generation cephalosporins, **TETRA**: Tetracyclines, **TGCEPH**: Third-generation cephalosporins, **XPLACB**: No antibiotics or Placebo

© 2026 Motaghi S et al. *JAMA Network Open*.

eTable 5. League Table for All-Cause Mortality—Relative Risk (95% CI)

**AMILIN**	0.25 (0.01,5.14)	0.62 (0.08,4.99)	0.62 (0.05,7.42)	23.50 (0.63,878.79)	0.44 (0.04,5.34)	0.84 (0.06,11.90)	0.95 (0.12,7.77)	1.89 (0.22,16.45)	5.60 (0.27,117.76)	1.06 (0.09,12.25)	3.05 (0.13,73.14)	0.91 (0.07,11.60)	1.83 (0.23,14.31)	1.59 (0.19,13.25)	0.87 (0.08,9.11)	1.29 (0.15,11.24)	1.01 (0.03,34.00)	1.44 (0.18,11.65)	1.35 (0.14,12.83)	1.12 (0.13,10.01)	3.01 (0.38,24.00)
3.93 (0.19,79.59)	**AMIMAC**	2.44 (0.23,26.09)	2.44 (0.14,43.85)	92.45 (1.88,4545.64)	1.71 (0.09,33.55)	3.32 (0.16,67.36)	3.75 (0.44,32.30)	7.42 (0.55,99.97)	22.03 (0.76,638.50)	4.18 (0.25,70.43)	11.99 (0.37,388.70)	3.58 (0.21,61.24)	7.21 (0.65,80.28)	6.25 (0.49,80.00)	3.42 (0.24,49.16)	5.08 (0.38,67.41)	3.96 (0.09,177.15)	5.69 (0.44,73.35)	5.30 (0.40,70.02)	4.41 (0.32,60.39)	11.82 (0.78,178.93)
1.61 (0.20,13.02)	0.41 (0.04,4.39)	**BRPEN**	1.00 (0.18,5.68)	37.94 (1.65,873.79)	0.70 (0.11,4.66)	1.36 (0.20,9.40)	1.54 (0.57,4.15)	3.05 (0.92,10.10)	9.04 (0.78,104.74)	1.71 (0.34,8.70)	4.92 (0.37,66.30)	1.47 (0.28,7.68)	2.96 (1.48,5.92)	2.56 (0.87,7.54)	1.40 (0.37,5.31)	2.09 (0.66,6.64)	1.63 (0.08,33.26)	2.33 (0.78,6.99)	2.18 (0.69,6.89)	1.81 (0.53,6.20)	4.85 (1.12,21.10)
1.61 (0.13,19.36)	0.41 (0.02,7.39)	1.00 (0.18,5.68)	**CARBA**	37.94 (1.35,1066.09)	0.70 (0.07,6.69)	1.36 (0.14,12.79)	1.54 (0.22,10.58)	3.05 (0.61,15.23)	9.04 (0.61,134.49)	1.71 (0.23,12.51)	4.92 (0.29,84.03)	1.47 (0.16,13.84)	2.96 (0.56,15.66)	2.56 (0.54,12.27)	1.40 (0.19,10.56)	2.09 (0.41,10.63)	1.63 (0.06,40.87)	2.33 (0.61,8.97)	2.18 (0.32,14.61)	1.81 (0.34,9.66)	4.85 (0.68,34.74)
0.04 (0.00,1.59)	**0.01 (0.00,0.53)**	**0.03 (0.00,0.61)**	**0.03 (0.00,0.74)**	**FGCEPH**	0.02 (0.00,0.53)	0.04 (0.00,0.92)	0.04 (0.00,1.04)	0.08 (0.00,1.85)	0.24 (0.01,8.53)	0.05 (0.00,1.09)	0.13 (0.00,5.74)	0.04 (0.00,1.20)	0.08 (0.00,1.71)	0.07 (0.00,1.30)	0.04 (0.00,1.00)	0.05 (0.00,1.08)	0.04 (0.00,2.31)	0.06 (0.00,1.30)	0.06 (0.00,1.44)	0.05 (0.00,0.85)	0.13 (0.00,3.32)
2.30 (0.19,28.22)	0.58 (0.03,11.45)	1.42 (0.21,9.44)	1.42 (0.15,13.56)	54.01 (1.90,1536.81)	**FLUORQ**	1.94 (0.20,18.56)	2.19 (0.28,17.08)	4.34 (0.65,29.01)	12.87 (0.85,194.45)	2.44 (0.30,20.02)	7.00 (0.37,131.56)	2.09 (0.20,21.87)	4.21 (0.69,25.60)	3.65 (0.65,20.64)	2.00 (0.24,16.87)	2.97 (0.51,17.42)	2.31 (0.09,58.94)	3.32 (0.54,20.24)	3.10 (0.41,23.51)	2.57 (0.47,14.09)	6.91 (1.27,37.54)
1.19 (0.08,16.76)	0.30 (0.01,6.13)	0.73 (0.11,5.08)	0.73 (0.08,6.91)	27.88 (1.09,716.24)	0.52 (0.05,4.95)	**FORTGC**	1.13 (0.14,9.28)	2.24 (0.32,15.44)	6.65 (0.50,88.40)	1.26 (0.17,9.41)	3.62 (0.20,64.03)	1.08 (0.10,11.73)	2.17 (0.34,13.88)	1.88 (0.37,9.62)	1.03 (0.12,9.08)	1.53 (0.29,8.03)	1.19 (0.05,27.37)	1.71 (0.29,10.28)	1.60 (0.20,12.68)	1.33 (0.30,5.89)	3.57 (0.43,29.76)
1.05 (0.13,8.56)	0.27 (0.03,2.30)	0.65 (0.24,1.75)	0.65 (0.09,4.47)	24.65 (0.96,633.28)	0.46 (0.06,3.56)	0.88 (0.11,7.26)	**METAMI**	1.98 (0.46,8.51)	5.88 (0.44,78.16)	1.11 (0.18,6.93)	3.20 (0.21,49.14)	0.95 (0.15,6.08)	1.92 (0.65,5.68)	1.67 (0.43,6.53)	0.91 (0.19,4.39)	1.36 (0.32,5.66)	1.06 (0.05,24.20)	1.52 (0.38,6.03)	1.41 (0.34,5.86)	1.18 (0.27,5.21)	3.15 (0.60,16.53)
0.53 (0.06,4.63)	0.13 (0.01,1.82)	0.33 (0.10,1.09)	0.33 (0.07,1.64)	12.46 (0.54,286.70)	0.23 (0.03,1.54)	0.45 (0.06,3.08)	0.51 (0.12,2.17)	**METBRO**	2.97 (0.26,34.36)	0.56 (0.11,2.88)	1.62 (0.12,21.79)	0.48 (0.08,3.03)	0.97 (0.34,2.80)	0.84 (0.29,2.48)	0.46 (0.10,2.18)	0.69 (0.21,2.20)	0.53 (0.03,10.91)	0.77 (0.32,1.85)	0.71 (0.18,2.91)	0.59 (0.17,2.03)	1.59 (0.36,7.11)
0.18 (0.01,3.76)	0.05 (0.00,1.32)	0.11 (0.01,1.28)	0.11 (0.01,1.64)	4.20 (0.12,150.23)	0.08 (0.01,1.17)	0.15 (0.01,2.00)	0.17 (0.01,2.26)	0.34 (0.03,3.90)	**METFGC**	0.19 (0.02,2.34)	0.54 (0.02,13.95)	0.16 (0.01,2.73)	0.33 (0.03,3.56)	0.28 (0.03,2.61)	0.16 (0.01,2.19)	0.23 (0.02,2.16)	0.18 (0.01,5.80)	0.26 (0.02,2.68)	0.24 (0.02,3.11)	0.20 (0.02,1.66)	0.54 (0.04,7.24)
0.94 (0.08,10.87)	0.24 (0.01,4.04)	0.58 (0.11,2.96)	0.58 (0.08,4.26)	22.13 (0.92,535.16)	0.41 (0.05,3.36)	0.79 (0.11,5.93)	0.90 (0.14,5.59)	1.78 (0.35,9.08)	5.28 (0.43,65.01)	**METMON**	2.87 (0.20,41.41)	0.86 (0.10,7.32)	1.73 (0.37,7.99)	1.50 (0.44,5.14)	0.82 (0.12,5.52)	1.22 (0.39,3.81)	0.95 (0.04,20.41)	1.36 (0.32,5.87)	1.27 (0.21,7.59)	1.06 (0.27,4.08)	2.83 (0.43,18.60)
0.33 (0.01,7.88)	0.08 (0.00,2.70)	0.20 (0.02,2.74)	0.20 (0.01,3.47)	7.71 (0.17,341.35)	0.14 (0.01,2.68)	0.28 (0.02,4.90)	0.31 (0.02,4.81)	0.62 (0.05,8.35)	1.84 (0.07,47.14)	0.35 (0.02,5.03)	**METPEN**	0.30 (0.02,5.72)	0.60 (0.05,7.64)	0.52 (0.05,5.56)	0.29 (0.02,4.62)	0.42 (0.04,4.74)	0.33 (0.01,13.27)	0.47 (0.04,5.77)	0.44 (0.03,6.61)	0.37 (0.03,4.30)	0.99 (0.06,15.74)
1.10 (0.09,14.03)	0.28 (0.02,4.78)	0.68 (0.13,3.56)	0.68 (0.07,6.42)	25.84 (0.83,801.80)	0.48 (0.05,5.01)	0.93 (0.09,10.08)	1.05 (0.16,6.68)	2.07 (0.33,13.03)	6.16 (0.37,103.45)	1.17 (0.14,9.98)	3.35 (0.17,64.21)	**METPHO**	2.01 (0.45,9.04)	1.75 (0.30,10.21)	0.96 (0.33,2.81)	1.42 (0.23,8.76)	1.11 (0.04,30.84)	1.59 (0.26,9.56)	1.48 (0.32,6.96)	1.23 (0.19,7.95)	3.31 (0.44,24.87)
0.55 (0.07,4.27)	0.14 (0.01,1.55)	**0.34 (0.17,0.68)**	0.34 (0.06,1.79)	12.83 (0.58,281.73)	0.24 (0.04,1.44)	0.46 (0.07,2.94)	0.52 (0.18,1.54)	1.03 (0.36,2.97)	3.06 (0.28,33.31)	0.58 (0.13,2.69)	1.66 (0.13,21.15)	0.50 (0.11,2.23)	**METRO**	0.87 (0.34,2.19)	0.47 (0.15,1.48)	0.71 (0.25,1.97)	0.55 (0.03,10.70)	0.79 (0.30,2.11)	0.74 (0.29,1.85)	0.61 (0.20,1.85)	1.64 (0.43,6.32)
0.63 (0.08,5.25)	0.16 (0.01,2.05)	0.39 (0.13,1.15)	0.39 (0.08,1.87)	14.79 (0.77,285.58)	0.27 (0.05,1.55)	0.53 (0.10,2.71)	0.60 (0.15,2.35)	1.19 (0.40,3.50)	3.53 (0.38,32.43)	0.67 (0.19,2.30)	1.92 (0.18,20.46)	0.57 (0.10,3.35)	1.15 (0.46,2.92)	**METSGC**	0.55 (0.13,2.37)	0.81 (0.51,1.30)	0.63 (0.04,10.79)	0.91 (0.41,2.02)	0.85 (0.23,3.14)	0.71 (0.36,1.37)	1.89 (0.45,7.98)
1.15 (0.11,12.05)	0.29 (0.02,4.20)	0.71 (0.19,2.70)	0.71 (0.09,5.35)	27.02 (1.00,726.30)	0.50 (0.06,4.22)	0.97 (0.11,8.53)	1.10 (0.23,5.27)	2.17 (0.46,10.25)	6.44 (0.46,90.66)	1.22 (0.18,8.23)	3.50 (0.22,56.74)	1.05 (0.36,3.07)	2.11 (0.68,6.56)	1.83 (0.42,7.92)	**METTET**	1.49 (0.32,6.86)	1.16 (0.05,27.80)	1.66 (0.37,7.46)	1.55 (0.48,4.99)	1.29 (0.26,6.28)	3.46 (0.59,20.15)
0.77 (0.09,6.74)	0.20 (0.01,2.61)	0.48 (0.15,1.53)	0.48 (0.09,2.44)	18.18 (0.93,356.06)	0.34 (0.06,1.97)	0.65 (0.12,3.42)	0.74 (0.18,3.08)	1.46 (0.45,4.69)	4.33 (0.46,40.63)	0.82 (0.26,2.57)	2.36 (0.21,26.35)	0.70 (0.11,4.34)	1.42 (0.51,3.95)	1.23 (0.77,1.97)	0.67 (0.15,3.11)	**METTGC**	0.78 (0.05,13.46)	1.12 (0.45,2.79)	1.04 (0.26,4.14)	0.87 (0.42,1.79)	2.33 (0.52,10.40)
0.99 (0.03,33.55)	0.25 (0.01,11.29)	0.62 (0.03,12.59)	0.62 (0.02,15.46)	23.34 (0.43,1260.45)	0.43 (0.02,11.01)	0.84 (0.04,19.18)	0.95 (0.04,21.69)	1.87 (0.09,38.29)	5.56 (0.17,179.60)	1.05 (0.05,22.69)	3.03 (0.08,121.53)	0.90 (0.03,25.16)	1.82 (0.09,35.42)	1.58 (0.09,26.85)	0.86 (0.04,20.75)	1.28 (0.07,22.17)	**PENIC**	1.44 (0.08,26.87)	1.34 (0.06,29.97)	1.11 (0.07,17.50)	2.99 (0.13,69.17)
0.69 (0.09,5.58)	0.18 (0.01,2.27)	0.43 (0.14,1.28)	0.43 (0.11,1.65)	16.26 (0.77,343.98)	0.30 (0.05,1.84)	0.58 (0.10,3.50)	0.66 (0.17,2.62)	1.31 (0.54,3.15)	3.88 (0.37,40.22)	0.73 (0.17,3.17)	2.11 (0.17,25.64)	0.63 (0.10,3.79)	1.27 (0.47,3.39)	1.10 (0.49,2.44)	0.60 (0.13,2.70)	0.89 (0.36,2.23)	0.70 (0.04,13.05)	**SGCEPH**	0.93 (0.24,3.59)	0.78 (0.29,2.10)	2.08 (0.49,8.74)
0.74 (0.08,7.06)	0.19 (0.01,2.49)	0.46 (0.15,1.46)	0.46 (0.07,3.09)	17.44 (0.69,438.02)	0.32 (0.04,2.45)	0.63 (0.08,4.96)	0.71 (0.17,2.93)	1.40 (0.34,5.70)	4.16 (0.32,53.75)	0.79 (0.13,4.71)	2.26 (0.15,33.79)	0.67 (0.14,3.17)	1.36 (0.54,3.42)	1.18 (0.32,4.36)	0.65 (0.20,2.08)	0.96 (0.24,3.81)	0.75 (0.03,16.73)	1.07 (0.28,4.12)	**TETRA**	0.83 (0.20,3.51)	2.23 (0.44,11.42)
0.89 (0.10,7.98)	0.23 (0.02,3.11)	0.55 (0.16,1.90)	0.55 (0.10,2.95)	20.98 (1.17,375.46)	0.39 (0.07,2.13)	0.75 (0.17,3.33)	0.85 (0.19,3.77)	1.68 (0.49,5.76)	5.00 (0.60,41.53)	0.95 (0.25,3.66)	2.72 (0.23,31.80)	0.81 (0.13,5.24)	1.64 (0.54,4.94)	1.42 (0.73,2.76)	0.78 (0.16,3.79)	1.15 (0.56,2.38)	0.90 (0.06,14.14)	1.29 (0.48,3.49)	1.20 (0.29,5.07)	**TGCEPH**	2.68 (0.59,12.17)
0.33 (0.04,2.66)	0.08 (0.01,1.28)	**0.21 (0.05,0.90)**	0.21 (0.03,1.47)	7.82 (0.30,203.02)	**0.14 (0.03,0.79)**	0.28 (0.03,2.34)	0.32 (0.06,1.66)	0.63 (0.14,2.80)	1.86 (0.14,25.13)	0.35 (0.05,2.32)	1.01 (0.06,16.18)	0.30 (0.04,2.28)	0.61 (0.16,2.35)	0.53 (0.13,2.23)	0.29 (0.05,1.69)	0.43 (0.10,1.92)	0.33 (0.01,7.76)	0.48 (0.11,2.02)	0.45 (0.09,2.30)	0.37 (0.08,1.69)	**XPLACB**

Node abbreviation: AMILIN: Aminoglycosides and Lincosamides, **BRPEN**: Broad-spectrum penicillins, **CARBA**: Carbapenems, **FGCEPH**: First-generation cephalosporin, **FORTGC**: Fourth-generation cephalosporin, **METAMI**: Aminoglycosides and Metronidazole, **METBRO**: Metronidazole and Broad-spectrum penicillins, **METPHO**: Metronidazole and Phosphonic acid, **METRO**: Metronidazole, **METSGC**: Metronidazole and Second-generation cephalosporins, **METTET**: Tetracyclines and Metronidazole, **METTGC**: Metronidazole and Third-generation cephalosporins, **SGGEPH**: Second-generation cephalosporins, **TETRA**: Tetracyclines, **TGCEPH**: Third-generation cephalosporins, **XPLACB**: No antibiotics or Placebo

© 2026 Motaghi S et al. *JAMA Network Open*.

eTable 6. League Table for Adverse Events—Relative Risk (95% CI)

**AMILIN**	3.04 (0.12,74.01)	1.96 (0.05,73.01)	2.83 (0.05,161.21)	1.12 (0.02,56.54)	1.57 (0.03,77.32)	0.30 (0.00,19.47)	3.01 (0.01,611.67)	4.36 (0.05,364.51)	2.96 (0.07,125.21)	2.84 (0.01,605.52)	4.10 (0.10,166.58)	1.17 (0.03,41.71)	2.38 (0.02,324.94)	1.37 (0.03,67.73)	1.29 (0.02,106.37)
0.33 (0.01,8.02)	**BRPEN**	0.65 (0.12,3.53)	0.93 (0.08,11.11)	0.37 (0.04,3.59)	0.52 (0.06,4.81)	0.10 (0.01,1.46)	0.99 (0.01,69.31)	1.44 (0.07,30.76)	0.98 (0.14,6.89)	0.93 (0.01,69.47)	1.35 (0.21,8.84)	0.38 (0.08,1.92)	0.78 (0.02,32.94)	0.45 (0.05,4.25)	0.42 (0.02,8.93)
0.51 (0.01,19.00)	1.55 (0.28,8.48)	**CARBA**	1.44 (0.20,10.44)	0.57 (0.07,4.92)	0.80 (0.08,7.78)	0.15 (0.02,1.45)	1.54 (0.03,78.79)	2.23 (0.16,30.34)	1.51 (0.53,4.31)	1.45 (0.03,79.43)	2.09 (0.86,5.05)	0.60 (0.33,1.08)	1.21 (0.04,35.95)	0.70 (0.08,5.80)	0.66 (0.05,9.06)
0.35 (0.01,20.13)	1.07 (0.09,12.80)	0.69 (0.10,5.01)	**FGCEPH**	0.40 (0.02,6.73)	0.56 (0.03,10.31)	0.10 (0.01,1.87)	1.06 (0.01,83.66)	1.54 (0.06,38.79)	1.05 (0.12,9.47)	1.00 (0.01,83.70)	1.45 (0.17,12.51)	0.41 (0.06,2.73)	0.84 (0.02,40.35)	0.48 (0.03,8.01)	0.46 (0.02,11.24)
0.89 (0.02,45.12)	2.71 (0.28,26.46)	1.75 (0.20,15.07)	2.53 (0.15,43.04)	**FORTGC**	1.41 (0.31,6.28)	0.26 (0.01,5.51)	2.69 (0.03,231.13)	3.90 (0.14,110.25)	2.65 (0.25,27.92)	2.54 (0.03,231.06)	3.66 (0.40,33.81)	1.04 (0.13,8.65)	2.13 (0.04,112.96)	1.22 (0.83,1.80)	1.15 (0.04,32.32)
0.64 (0.01,31.18)	1.93 (0.21,17.91)	1.24 (0.13,12.05)	1.80 (0.10,33.30)	0.71 (0.16,3.17)	**METAMI**	0.19 (0.01,4.24)	1.91 (0.02,173.88)	2.77 (0.09,84.52)	1.88 (0.16,22.11)	1.80 (0.02,173.69)	2.60 (0.25,27.34)	0.74 (0.08,6.88)	1.51 (0.03,85.53)	0.87 (0.20,3.68)	0.82 (0.03,24.74)
3.38 (0.05,222.77)	10.27 (0.68,154.25)	6.63 (0.69,63.55)	9.57 (0.53,171.37)	3.79 (0.18,78.99)	5.33 (0.24,120.26)	**METBRO**	10.19 (0.11,916.28)	14.76 (0.49,444.53)	10.02 (0.86,117.08)	9.60 (0.10,915.13)	13.85 (1.23,155.42)	3.95 (0.45,35.03)	8.05 (0.14,449.36)	4.62 (0.23,94.09)	4.36 (0.15,128.97)
0.33 (0.00,67.36)	1.01 (0.01,70.41)	0.65 (0.01,33.34)	0.94 (0.01,73.78)	0.37 (0.00,31.89)	0.52 (0.01,47.48)	0.10 (0.00,8.82)	**METPHO**	1.45 (0.06,33.36)	0.98 (0.02,48.29)	0.94 (0.15,6.08)	1.36 (0.02,75.32)	0.39 (0.01,19.84)	0.79 (0.03,20.83)	0.45 (0.01,38.29)	0.43 (0.01,18.04)
0.23 (0.00,19.16)	0.70 (0.03,14.91)	0.45 (0.03,6.12)	0.65 (0.03,16.32)	0.26 (0.01,7.26)	0.36 (0.01,11.01)	0.07 (0.00,2.04)	0.69 (0.03,15.92)	**METRO**	0.68 (0.05,8.48)	0.65 (0.02,17.08)	0.94 (0.06,14.35)	0.27 (0.02,3.66)	0.55 (0.04,8.15)	0.31 (0.01,8.67)	0.30 (0.02,3.80)
0.34 (0.01,14.27)	1.03 (0.15,7.24)	0.66 (0.23,1.89)	0.96 (0.11,8.64)	0.38 (0.04,3.99)	0.53 (0.05,6.25)	0.10 (0.01,1.17)	1.02 (0.02,49.98)	1.47 (0.12,18.38)	**METSGC**	0.96 (0.02,50.62)	1.38 (0.38,5.00)	0.39 (0.13,1.23)	0.80 (0.03,23.18)	0.46 (0.05,4.71)	0.44 (0.03,6.25)
0.35 (0.00,75.17)	1.07 (0.01,79.56)	0.69 (0.01,37.88)	1.00 (0.01,83.22)	0.39 (0.00,35.94)	0.55 (0.01,53.47)	0.10 (0.00,9.93)	1.06 (0.16,6.85)	1.54 (0.06,40.36)	1.04 (0.02,55.14)	**METTET**	1.44 (0.02,85.51)	0.41 (0.01,22.52)	0.84 (0.04,19.54)	0.48 (0.01,43.16)	0.45 (0.01,19.77)
0.24 (0.01,9.93)	0.74 (0.11,4.86)	0.48 (0.20,1.16)	0.69 (0.08,5.97)	0.27 (0.03,2.53)	0.38 (0.04,4.04)	**0.07 (0.01,0.81)**	0.74 (0.01,40.78)	1.07 (0.07,16.28)	0.72 (0.20,2.61)	0.69 (0.01,41.07)	**METTGC**	0.29 (0.10,0.81)	0.58 (0.02,18.88)	0.33 (0.04,2.98)	0.31 (0.02,4.91)
0.86 (0.02,30.51)	2.60 (0.52,12.95)	1.68 (0.93,3.03)	2.42 (0.37,15.99)	0.96 (0.12,7.93)	1.35 (0.15,12.48)	0.25 (0.03,2.24)	2.58 (0.05,131.78)	3.73 (0.27,51.00)	2.53 (0.82,7.87)	2.43 (0.04,132.71)	3.50 (1.24,9.93)	**SGCEPH**	2.04 (0.07,59.74)	1.17 (0.15,9.35)	1.10 (0.08,14.71)
0.42 (0.00,57.36)	1.28 (0.03,53.63)	0.82 (0.03,24.38)	1.19 (0.02,57.05)	0.47 (0.01,24.98)	0.66 (0.01,37.43)	0.12 (0.00,6.93)	1.27 (0.05,33.39)	1.83 (0.12,27.37)	1.24 (0.04,35.90)	1.19 (0.05,27.79)	1.72 (0.05,55.89)	0.49 (0.02,14.41)	**TETRA**	0.57 (0.01,29.93)	0.54 (0.03,9.95)
0.73 (0.01,36.26)	2.22 (0.24,20.97)	1.43 (0.17,11.92)	2.07 (0.12,34.33)	0.82 (0.56,1.21)	1.15 (0.27,4.89)	0.22 (0.01,4.40)	2.20 (0.03,186.14)	3.19 (0.12,88.29)	2.17 (0.21,22.15)	2.08 (0.02,186.12)	3.00 (0.34,26.76)	0.86 (0.11,6.84)	1.74 (0.03,90.78)	**TGCEPH**	0.94 (0.03,25.88)
0.78 (0.01,64.06)	2.36 (0.11,49.60)	1.52 (0.11,20.96)	2.20 (0.09,54.22)	0.87 (0.03,24.39)	1.22 (0.04,36.94)	0.23 (0.01,6.79)	2.34 (0.06,98.67)	3.39 (0.26,43.52)	2.30 (0.16,33.03)	2.20 (0.05,95.92)	3.18 (0.20,49.64)	0.91 (0.07,12.11)	1.85 (0.10,33.94)	1.06 (0.04,29.11)	**XPLACB**

Node abbreviation: AMILIN: Aminoglycosides and Lincosamides, **BRPEN**: Broad-spectrum penicillins, **CARBA**: Carbapenems, **FGCEPH**: First-generation cephalosporin, **FORTGC**: Fourth-generation cephalosporin, **METAMI**: Aminoglycosides and Metronidazole, **METBRO**: Metronidazole and Broad-spectrum penicillins, **METPHO**: Metronidazole and Phosphonic acid, **METRO**: Metronidazole, **METSGC**: Metronidazole and Second-generation cephalosporins, **METTET**: Tetracyclines and Metronidazole, **METTGC**: Metronidazole and Third-generation cephalosporins, **SGGEPH**: Second-generation cephalosporins, **TETRA**: Tetracyclines, **TGCEPH**: Third-generation cephalosporins, **XPLACB**: No antibiotics or Placebo

© 2026 Motaghi S et al. *JAMA Network Open*.

eTable 7. League Table for Length of Hospital Stay—Mean Difference (95% CI)

**AMILIN**	0.86 (-18.56,20.28)	2.92 (-8.25,14.09)	7.82 (-13.05,28.70)	-1.00 (-13.27,11.27)	5.86 (-8.06,19.78)	7.20 (-11.79,26.19)	12.80 (-9.88,35.48)	10.50 (-12.35,33.34)	8.80 (-10.07,27.67)	6.40 (-19.57,32.36)	6.20 (-12.70,25.09)	1.71 (-15.44,18.86)	7.20 (-7.03,21.42)	8.71 (-2.94,20.37)
-0.86 (-20.28,18.56)	**AMIMAC**	2.06 (-15.57,19.69)	6.96 (-16.37,30.29)	-1.86 (-24.84,21.11)	5.00 (-8.54,18.54)	6.33 (-15.32,27.99)	11.93 (-13.02,36.89)	9.63 (-15.47,34.74)	7.93 (-13.62,29.49)	5.53 (-22.44,33.51)	5.33 (-16.24,26.91)	0.85 (-22.40,24.10)	6.33 (-11.29,23.96)	7.85 (-11.71,27.40)
-2.92 (-14.09,8.25)	-2.06 (-19.69,15.57)	**BRPEN**	4.90 (-15.79,25.59)	-3.92 (-20.52,12.67)	2.94 (-8.35,14.23)	4.27 (-14.51,23.05)	9.87 (-12.63,32.38)	7.57 (-15.10,30.25)	5.87 (-12.79,24.53)	3.47 (-22.34,29.29)	3.27 (-15.41,21.96)	-1.21 (-20.04,17.62)	4.27 (-9.67,18.22)	5.79 (-8.22,19.80)
-7.82 (-28.70,13.05)	-6.96 (-30.29,16.37)	-4.90 (-25.59,15.79)	**CARBA**	-8.82 (-33.04,15.39)	-1.96 (-20.96,17.04)	-0.63 (-20.42,19.17)	4.97 (-10.47,20.41)	2.67 (-13.01,18.35)	0.97 (-7.97,9.92)	-1.43 (-21.38,18.53)	-1.63 (-21.33,18.08)	-6.11 (-29.20,16.98)	-0.63 (-15.92,14.66)	0.89 (-18.47,20.25)
1.00 (-11.27,13.27)	1.86 (-21.11,24.84)	3.92 (-12.67,20.52)	8.82 (-15.39,33.04)	**LINMON**	6.86 (-11.70,25.42)	8.20 (-14.41,30.81)	13.80 (-11.99,39.58)	11.50 (-14.44,37.43)	9.80 (-12.71,32.31)	7.40 (-21.32,36.12)	7.20 (-15.34,29.73)	2.71 (-18.38,23.80)	8.20 (-10.59,26.98)	9.71 (-7.21,26.64)
-5.86 (-19.78,8.06)	-5.00 (-18.54,8.54)	-2.94 (-14.23,8.35)	1.96 (-17.04,20.96)	-6.86 (-25.42,11.70)	**METAMI**	1.33 (-15.56,18.23)	6.93 (-14.03,27.90)	4.63 (-16.51,25.77)	2.93 (-13.83,19.70)	0.53 (-23.95,25.01)	0.33 (-16.46,17.13)	-4.15 (-23.05,14.75)	1.33 (-9.95,12.62)	2.85 (-11.26,16.96)
-7.20 (-26.19,11.79)	-6.33 (-27.99,15.32)	-4.27 (-23.05,14.51)	0.63 (-19.17,20.42)	-8.20 (-30.81,14.41)	-1.33 (-18.23,15.56)	**METFGC**	5.60 (-16.09,27.29)	3.30 (-18.56,25.16)	1.60 (-16.06,19.26)	-0.80 (-25.90,24.30)	-1.00 (-18.69,16.69)	-5.48 (-26.89,15.92)	-0.00 (-12.58,12.58)	1.52 (-15.80,18.83)
-12.80 (-35.48,9.88)	-11.93 (-36.89,13.02)	-9.87 (-32.38,12.63)	-4.97 (-20.41,10.47)	-13.80 (-39.58,11.99)	-6.93 (-27.90,14.03)	-5.60 (-27.29,16.09)	**METPEN**	-2.30 (-20.31,15.71)	-4.00 (-16.58,8.58)	-6.40 (-28.23,15.43)	-6.60 (-28.20,15.00)	-11.08 (-35.82,13.65)	-5.60 (-23.27,12.07)	-4.08 (-25.38,17.21)
-10.50 (-33.34,12.35)	-9.63 (-34.74,15.47)	-7.57 (-30.25,15.10)	-2.67 (-18.35,13.01)	-11.50 (-37.43,14.44)	-4.63 (-25.77,16.51)	-3.30 (-25.16,18.56)	2.30 (-15.71,20.31)	**METRO**	-1.70 (-14.58,11.18)	-4.10 (-16.44,8.24)	-4.30 (-26.08,17.48)	-8.78 (-33.67,16.10)	-3.30 (-21.18,14.58)	-1.78 (-23.26,19.69)
-8.80 (-27.67,10.07)	-7.93 (-29.49,13.62)	-5.87 (-24.53,12.79)	-0.97 (-9.92,7.97)	-9.80 (-32.31,12.71)	-2.93 (-19.70,13.83)	-1.60 (-19.26,16.06)	4.00 (-8.58,16.58)	1.70 (-11.18,14.58)	**METSGC**	-2.40 (-20.24,15.44)	-2.60 (-20.16,14.96)	-7.08 (-28.38,14.21)	-1.60 (-14.00,10.80)	-0.08 (-17.26,17.10)
-6.40 (-32.36,19.57)	-5.53 (-33.51,22.44)	-3.47 (-29.29,22.34)	1.43 (-18.53,21.38)	-7.40 (-36.12,21.32)	-0.53 (-25.01,23.95)	0.80 (-24.30,25.90)	6.40 (-15.43,28.23)	4.10 (-8.24,16.44)	2.40 (-15.44,20.24)	**METTET**	-0.20 (-25.23,24.83)	-4.68 (-32.46,23.10)	0.80 (-20.92,22.52)	2.32 (-22.45,27.08)
-6.20 (-25.09,12.70)	-5.33 (-26.91,16.24)	-3.27 (-21.96,15.41)	1.63 (-18.08,21.33)	-7.20 (-29.73,15.34)	-0.33 (-17.13,16.46)	1.00 (-16.69,18.69)	6.60 (-15.00,28.20)	4.30 (-17.48,26.08)	2.60 (-14.96,20.16)	0.20 (-24.83,25.23)	**METTGC**	-4.48 (-25.80,16.84)	1.00 (-11.44,13.44)	2.52 (-14.69,19.72)
-1.71 (-18.86,15.44)	-0.85 (-24.10,22.40)	1.21 (-17.62,20.04)	6.11 (-16.98,29.20)	-2.71 (-23.80,18.38)	4.15 (-14.75,23.05)	5.48 (-15.92,26.89)	11.08 (-13.65,35.82)	8.78 (-16.10,33.67)	7.08 (-14.21,28.38)	4.68 (-23.10,32.46)	4.48 (-16.84,25.80)	**TETRA**	5.48 (-11.83,22.80)	7.00 (-5.58,19.58)
-7.20 (-21.42,7.03)	-6.33 (-23.96,11.29)	-4.27 (-18.22,9.67)	0.63 (-14.66,15.92)	-8.20 (-26.98,10.59)	-1.33 (-12.62,9.95)	0.00 (-12.58,12.58)	5.60 (-12.07,23.27)	3.30 (-14.58,21.18)	1.60 (-10.80,14.00)	-0.80 (-22.52,20.92)	-1.00 (-13.44,11.44)	-5.48 (-22.80,11.83)	**TGCEPH**	1.52 (-10.38,13.41)
-8.71 (-20.37,2.94)	-7.85 (-27.40,11.71)	-5.79 (-19.80,8.22)	-0.89 (-20.25,18.47)	-9.71 (-26.64,7.21)	-2.85 (-16.96,11.26)	-1.52 (-18.83,15.80)	4.08 (-17.21,25.38)	1.78 (-19.69,23.26)	0.08 (-17.10,17.26)	-2.32 (-27.08,22.45)	-2.52 (-19.72,14.69)	-7.00 (-19.58,5.58)	-1.52 (-13.41,10.38)	**XPLACB**

Node abbreviation: AMILIN: Aminoglycosides and Lincosamides, **AMIMAC**: Aminoglycosides and Macrolides, **BRPEN**: Broad-spectrum penicillins, **CARBA**: Carbapenems, **LINMON**: Lincosamides and Monobactams, **METAMI**: Aminoglycosides and Metronidazole, **METFGC**: Metronidazole and First-generation cephalosporins, **METPEN**: Metronidazole and Penicillin, **METRO**: Metronidazole, **METSGC**: Metronidazole and Second-generation cephalosporins, **METTET**: Tetracyclines and Metronidazole, **METTGC**: Metronidazole and Third-generation cephalosporins, **TETRA**: Tetracyclines, **TGCEPH**: Third-generation cephalosporins, **XPLACB**: No antibiotics or Placebo

© 2026 Motaghi S et al. *JAMA Network Open*.

eTable 8. Subgroup Analysis for Surgical Site Infection—Low Risk of Bias vs High Risk of Bias

Antibiotic class/Combination		β Coefficient	95% CI		P>z
Aminoglycosides and Lincosamides	test of interaction	-0.95	-2.97	1.07	0.35
	Low RoB	-0.5	-2.4	1.38	0.6
Aminoglycosides and Macrolides	test of interaction	.	.	.	.
	Low RoB	-1.22	-2.41	-0.03	0.04
Aminoglycosides	test of interaction	.	.	.	.
	Low RoB	0.19	-1579.04	1579.43	1
Broad-spectrum β-lactams	test of interaction	0.95	-0.7	2.61	0.26
	Low RoB	-2.3	-3.84	-0.75	0
Carbapenems	test of interaction	0.55	-1.37	2.47	0.57
	Low RoB	-2.09	-3.66	-0.52	0
First-generation cephalosporin	test of interaction	1.28	-1.77	4.34	0.41
	Low RoB	-2.59	-5.55	0.36	0.08
Fluoroquinolones	test of interaction	.	.	.	.
	Low RoB	-0.49	-1.33	0.34	0.25
Fourth-generation cephalosporin	test of interaction	.	.	.	.
	Low RoB	-1.34	-2.49	-0.18	0.02
Lincosamides	test of interaction	.	.	.	.
	Low RoB	-1.14	-2.44	0.16	0.08
Lincosamides and Monobactams	test of interaction	-1.95	-1581.19	1577.28	0.99
	Low RoB	-0.26	-1579.49	1578.97	1
Metronidazole and Second-generation cephalosporins and Aminoglycosides	test of interaction	.	.	.	.
	Low RoB	-1.28	-4.25	1.68	0.39
Aminoglycosides and Metronidazole	test of interaction	0.84	-0.65	2.34	0.27
	Low RoB	-1.67	-3.02	-0.32	0.01
Metronidazole and Broad-spectrum β-lactams	test of interaction	.	.	.	.
	Low RoB	-1.39	-2.16	-0.63	0
Metronidazole and First-generation cephalosporins	test of interaction	2.82	-1.08	6.74	0.15
	Low RoB	-3.66	-7.19	-0.14	0.04
Metronidazole and Fluoroquinolones	test of interaction	.	.	.	.
	Low RoB	-1.18	-3.75	1.38	0.37
Metronidazole and Monobactams	test of interaction	.	.	.	.
	Low RoB	-1.17	-3.13	0.80	0.24
Metronidazole, Penicillin, and Aminoglycoside	test of interaction	.	.	.	.
	Low RoB	-1.09	-3.00	0.81	0.26
Metronidazole and Penicillin	test of interaction	.	.	.	.
	Low RoB	-0.79	-1.92	0.34	0.17
Metronidazole and Phosphonic acid	test of interaction	.	.	.	.
	Low RoB	-2.88	-5.27	-0.48	0.02
Metronidazole	test of interaction	0.68	-0.50	1.85	0.26

© 2026 Motaghi S et al. *JAMA Network Open*.

	Low RoB	-1.45	-2.52	-0.38	0.01
Metronidazole and Second-generation cephalosporins	test of interaction	0.84	-0.85	2.54	0.33
	Low RoB	-2.16	-3.76	-0.57	0.01
Metronidazole and Sulfonamide	test of interaction	.	.	.	.
	Low RoB	-1.50	-3.37	0.37	0.12
Tetracyclines and Metronidazole	test of interaction	0.11	-2.46	2.68	0.93
	Low RoB	-1.79	-4.21	0.63	0.15
Metronidazole and Third-generation cephalosporins	test of interaction	0.82	-0.99	2.62	0.38
	Low RoB	-2.09	-3.74	-0.44	0.01
Metronidazole and Monobactam	test of interaction	.	.	.	.
	Low RoB	-1.72	-3.71	0.27	0.09
Penicillin, Aminoglycosides, and Lincosamide	test of interaction	.	.	.	.
	Low RoB	-1.59	-4.13	0.95	0.22
Penicillin and Aminoglycosides	test of interaction	.	.	.	.
	Low RoB	-0.35	-1.90	1.20	0.66
Penicillin	test of interaction	.	.	.	.
	Low RoB	-0.94	-1.72	-0.16	0.02
Second-generation cephalosporins	test of interaction	0.51	-1.09	2.11	0.53
	Low RoB	-1.64	-3.14	-0.14	0.03
Tetracyclines	test of interaction	-0.23	-1.41	0.95	0.70
	Low RoB	-0.99	-1.99	0.00	0.05
Third-generation cephalosporins	test of interaction	0.64	-1.11	2.38	0.48
	Low RoB	-1.98	-3.62	-0.35	0.02

© 2026 Motaghi S et al. *JAMA Network Open*.

eReferences.

1. Aberg C, Olin B, Oresland T, et al. Comparison of metronidazole with doxycycline prophylaxis in elective colorectal surgery. A prospective, randomized multicentre study. *Acta Chirurgica Scandinavica*. 1984;150(1):79-83.
2. AhChong K, Yip AW, Lee FC, Chiu KM. Comparison of prophylactic ampicillin/sulbactam with gentamicin and metronidazole in elective colorectal surgery: a randomized clinical study. *Journal of Hospital Infection*. 1994;27(2):149-154. doi:10.1016/0195-6701(94)90008-6
3. Ambrose NS, Donovan IA, Wise R, Lowe P. Metronidazole and ticarcillin in the prevention of sepsis after appendicectomy. *American Journal of Surgery*. 1983;146(3):346-8.
4. Andåker L, Burman LG, Eklund A, et al. Fosfomycin/metronidazole compared with doxycycline/metronidazole for the prophylaxis of infection after elective colorectal surgery. A randomised double-blind multicentre trial in 517 patients. *European journal of surgery = Acta chirurgica*. 1992;158(3):181-185.
5. Antonelli W, Borgani A, Machella C, et al. Comparison of two systemic antibiotics for the prevention of complications in elective colorectal surgery. *The italian journal of surgical sciences*. 1985;15(3):255-258.
6. Arnaud JP, Bellissant E, Boissel P, Carlet J, Chastang C, Lafaix C. Single-dose amoxycillin-clavulanic acid vs cefotetan for prophylaxis in elective colorectal surgery: a multicentre, prospective, randomized study. *Journal of Hospital Infection*. 1992;(22 Suppl A):23-32.
7. Barber MS, Hirschberg BC, Rice CL, Atkins CC. Parenteral antibiotics in elective colon surgery? A prospective, controlled clinical study. *Surgery*. 1979;86(1):23-9.
8. Bell GA, Fothergill J, Murphy J, Smith JA. Intravenous prophylactic antimicrobial drugs in elective colorectal operations. *Surgery, Gynecology & Obstetrics*. 1983;156(3):351-354.
9. Bellantone R, Pacelli F, Sofo L, et al. Systemic perioperative prophylaxis in elective oncological colorectal surgery: cefotetan versus clindamicin plus aztreonam. *Drugs Exp Clin Res*. 1988;14(12):763-6.
10. Blair JE, McLeod RS, Cohen Z, Devlin HR. Ticarcillin/clavulanic acid (Timentin) compared to metronidazole/netilmicin in preventing postoperative infection after elective colorectal surgery. *Canadian journal of surgery Journal canadien de chirurgie*. 1987;30(2):120-122.
11. Brolin J, Lahnborg G, Ljung A, Rietz KA. A comparison between netilmicin with metronidazole and doxycycline as prophylaxis in elective colorectal surgery. *Annales Chirurgiae et Gynaecologiae*. 1986;75(4):219-224.
12. Cainzos M, Potel J, Puente J. Short-term antibiotic prophylaxis in colorectal surgery: a comparative study of gentamicin plus clindamycin vs cefoxitin. *Acta therapeutica*. 1986;12(4):399-412.
13. Cunliffe WJ, Carr N, Schofield PF. Prophylactic metronidazole with and without cefuroxime in elective colorectal surgery. A prospective randomised double-blind study. *Journal of the Royal College of Surgeons of Edinburgh*. 1985;30(2):123-125.
14. Cuthbertson A, Ross H, Allsop J. Clinical trial of prophylaxis of wound sepsis in elective colorectal surgery. Cephamandole with tinidazole versus tinidazole alone. *Med J Aust*. Oct 29 1983;2(9):440-3.
15. de la Hunt MN, Karran SJ. Sulbactam/ampicillin compared with cefoxitin for chemoprophylaxis in elective colorectal surgery. *Diseases of the Colon and Rectum*. 1986;29(3):157-159. doi:10.1007/BF02555011
16. Diamond T, Mulholland CK, Hanna WA, Parks TG. A prospective randomized trial to compare triple dose mezlocillin with triple dose cefuroxime plus metronidazole as prophylaxis in colorectal surgery. *Journal of Hospital Infection*. 1988;12(3):215-219. doi:10.1016/0195-6701(88)90009-6
17. Eykyn SJ, Jackson BT, Lockhart-Mummery HE, Phillips I. Prophylactic peroperative intravenous metronidazole in elective colorectal surgery. *Lancet*. 1979;2(8146):761-4.
18. Favre JP, Bouchet Y, Clotteau JE, et al. Prophylactic use of cefotaxime in colonic and rectal surgery. *J Antimicrob Chemother*. Sep 1984;14 Suppl B:247-53. doi:10.1093/jac/14.suppl_b.247

© 2026 Motaghi S et al. *JAMA Network Open*.

19. Figueras-Felip J, Basilio-Bonet E, Lara-Eisman F, et al. Oral is superior to systemic antibiotic prophylaxis in operations upon the colon and rectum. *Surgery, Gynecology & Obstetrics*. 1984;158(4):359-362.
20. Gerner T, Nygaard K, Kaaresen R, Mjoerud J, Larsen S. Antibiotic prophylaxis in colorectal surgery. Combined doxycycline-tinidazole vs. doxycycline alone. *Acta Chirurgica Scandinavica*. 1989;155(2):121-4.
21. Görtz G, Boese-Landgraf J, Hopfenmüller W, et al. Ciprofloxacin as single-dose antibiotic prophylaxis in colorectal surgery results of a randomized, double-blind trial. *Diagnostic microbiology and infectious disease*. 1990;13(2):181-185. doi:10.1016/0732-8893%2890%2990106-6
22. Gottrup F, Diederich P, Sørensen K, Nielsen SV, Ornsholt J, Brandsborg O. Prophylaxis with whole gut irrigation and antimicrobials in colorectal surgery. A prospective, randomized double-blind clinical trial. *American Journal of Surgery*. 1985;149(3):317-322. doi:10.1016/s0002-9610(85)80098-2
23. Grüner OP, Holter O, Baardsen A. Combined tinidazole and doxycycline prophylaxis in colorectal surgery. An interhospital trial. *Scand J Gastroenterol Suppl*. 1980;59:25-8.
24. Hagen TB, Bergan T, Liavag I. Prophylactic metronidazole in elective colo-rectal surgery. *Acta Chirurgica Scandinavica*. 1980;146(1):71-75.
25. Håkansson T, Raahave D, Hansen OH, Pedersen T. Effectiveness of single dose prophylaxis with cefotaxime and metronidazole compared with three doses of cefotaxime alone in elective colorectal surgery. *European journal of surgery = Acta chirurgica*. 1993;159(3):177-180.
26. Hall C, Curran F, Burdon DW, Keighley MR. A randomized trial to compare amoxycillin/clavulanate with metronidazole plus gentamicin in prophylaxis in elective colorectal surgery. *Journal of Antimicrobial Chemotherapy*. 1989;24 Suppl B:195-202. doi:10.1093/jac/24.suppl_b.195
27. Haverkorn MJ. Peroperative systemic prophylaxis in colorectal surgery. *Drugs under Experimental and Clinical Research*. 1985;11(2):111-114.
28. Hershman MJ, Swift RI, Reilly DT, et al. Prospective comparative study of cefotetan with piperacillin for prophylaxis against infection in elective colorectal surgery. *Journal of the Royal College of Surgeons of Edinburgh*. 1990;35(1):29-32.
29. Hinchey EJ, Richards GK, Lewis R, Echave V, Biron JS, Weissglass I. Moxalactam as single-agent prophylaxis in the prevention of wound infection following colon surgery. *Surgery*. Jan 1987;101(1):15-9.
30. Hjalmarsson C, Karlberg J, Tornqvist P, Arbman G, Frisk B, Modin M. Orally Administered Trimethoprim-Sulfamethoxazole and Metronidazole as Infection Prophylaxis in Elective Colorectal Surgery. *Surgical Infections*. 2015;16(5):604-610. doi:https://dx.doi.org/10.1089/sur.2014.059
31. Hoffmann CEJ, McDonald PJ, Watts JM. Use of peroperative Cefoxitin(Reg.trademark) to prevent infection after colonic and rectal surgery. *Annals of Surgery*. 1981;193(3):353-356.
32. Höjer H. The effect on total antimicrobial consumption and hospitalization time after prophylactic treatment with doxycycline in colorectal surgery. *Acta Chir Scand*. 1978;144(3):175-9.
33. Höjer H, Bröte L, Nyström PO, Wetterfors J. Systemic prophylaxis in colorectal surgery a comparison between tinidazole and doxycycline. *Scandinavian journal of infectious diseases Supplementum*. 1981;26:75-78.
34. Hunt PS, Francis JK, Peck G, Farrell K, Sali A. Tinidazole in the prevention of wound infection after elective colorectal surgery. *Med J Aust*. Feb 24 1979;1(4):107-9. doi:10.5694/j.1326-5377.1979.tb112039.x
35. Itani KM, Wilson SE, Awad SS, Jensen EH, Finn TS, Abramson MA. Ertapenem versus cefotetan prophylaxis in elective colorectal surgery. *New England Journal of Medicine*. 2006;355(25):2640-51.
36. Ivarsson L, Darle N, Kewenter JG, Seeberg S, Norrby R. Short-term systemic prophylaxis with cefoxitin and doxycycline in colorectal surgery. A prospective, randomized study. *American Journal of Surgery*. 1982;144(2):257-261. doi:10.1016/0002-9610(82)90521-9
37. Jagelman DG, Fazio VW, Lavery IC, Weakley FL. A prospective, randomized, double-blind study of 10% mannitol mechanical bowel preparation combined with oral neomycin and short-term,

perioperative, intravenous Flagyl as prophylaxis in elective colorectal resections. *Surgery*. 1985;98(5):861-865.
38. Jagelman DG, Fazio VW, Lavery IC, Weakley FL, Chaney TL. A prospective randomized study of prophylactic mannitol (10%)-neomycin-cefotaxime therapy in patients undergoing elective colonic and rectal surgery. *Clinical Therapeutics*. 1982;5(Suppl. A):32-37.
39. Jagelman DG, Fazio VW, Lavery IC, Weakley FL, Tusek D. Single-dose piperacillin versus cefoxitin combined with 10 percent mannitol bowel preparation as prophylaxis in elective colorectal operations. *American Journal of Surgery*. 1987;154(5):478-481. doi:10.1016/0002-9610(87)90257-1
40. Jones RN, Wojeski WV. Single-dose cephalosporin prophylaxis of 929 surgical procedures in a prepaid group practice: a prospective, randomized comparison of cefoperazone and cefotaxime. *Diagn Microbiol Infect Dis*. Apr 1987;6(4):323-34. doi:10.1016/0732-8893(87)90183-0
41. Karran SJ, Sutton G, Gartell P, Karran SE, Finnis D, Blenkinsop J. Imipenem prophylaxis in elective colorectal surgery. *British Journal of Surgery*. 1993;80(9):1196-1198. doi:10.1002/bjs.1800800946
42. Keighley MR, Ambrose NS, Morris DL, Burdon DW. Evaluation of mezlocillin in elective gastrointestinal surgery. *J Antimicrob Chemother*. May 1983;11 Suppl C:65-9. doi:10.1093/jac/11.suppl_c.65
43. Keighley MR, Crapp AR, Burdon DW, Cooke WT, Alexander-Williams J. Prophylaxis against anaerobic sepsis in bowel surgery. *British Journal of Surgery*. 1976;63(7):538-41.
44. Kingston RD, Kiff RS, Duthie JS, Walsh S, Spicer A, Jeacock J. Comparison of two prophylactic single-dose intravenous antibiotic regimes in the treatment of patients undergoing elective colorectal surgery in a district general hospital. *J R Coll Surg Edinb*. Aug 1989;34(4):208-11.
45. Kling PA, Holmlund D, Burman LG. Single-dose intravenous metronidazole v. doxycycline prophylaxis in colorectal surgery. An open prospective, randomized trial. *Acta Chirurgica Scandinavica*. 1985;151(2):163-168.
46. Kwok SP, Lau WY, Leung KL, Ku KW, Ho WS, Li AK. Amoxycillin and clavulanic acid versus cefotaxime and metronidazole as antibiotic prophylaxis in elective colorectal resectional surgery. *CHEMOTHERAPY*. 1993;39(2):135-139. doi:10.1159/000239116
47. Lauridsen F, Bjoernsen K, Nielsen SA, Hart Hansen O. Short-term prophylaxis with cefotaxime in colorectal surgery. A prospective, randomized trial. *Dis Colon Rectum*. Jan 1988;31(1):25-7. doi:10.1007/bf02552565
48. Leandoer L, Ekelund G, Genell S, Olson S. Antibiotic prophylaxis in colorectal surgery. doxycycline compared to a combination of benzylpenicillin and streptomycin. A preliminary report. *Scand J Infect Dis Suppl*. 1976;(9):106-8.
49. Leng X, Zhao Y, Qiu H, et al. Ertapenem prophylaxis of surgical site infections in elective colorectal surgery in China: a multicentre, randomized, double-blind, active-controlled study. *The Journal of antimicrobial chemotherapy*. 2014;69(12):3379-3386. doi:https://dx.doi.org/10.1093/jac/dku302
50. Lindhagen J, Andaker L, Hojer H. Comparison of systemic prophylaxis with metronidazole/placebo and metronidazole/fosfomycin in colorectal surgery. A clinical study demonstrating the need for additional anti-aerobic prophylactic cover. *Acta Chirurgica Scandinavica*. 1984;150(4):317-323.
51. Lozano F, Gomez Alonso A, Almazan A. A comparison of three different prophylactic parenteral antibiotic regimens in colorectal surgery: A prospective study. *International Surgery*. 1985;70(3):227-231.
52. Luke M, Iversen J, Sondergaard J, et al. Ceftriaxone vs. ampicillin + metronidazole as prophylaxis against infections after clean-contaminated abdominal surgery. *Acta Chirurgica - European Journal of Surgery*. 1991;157(1):45-49.
53. Lumley JW, Siu SK, Pillay SP, et al. Single dose ceftriaxone as prophylaxis for sepsis in colorectal surgery. *Australian and New Zealand Journal of Surgery*. 1992;62(4):292-296.
54. Matikainen M, Hiltunen KM. Parenteral single dose ceftriaxone with tinidatsole versus aminoglycoside with tinidatsole in colorectal surgery: a prospective single-blind randomized multicentre study. *Int J Colorectal Dis*. Sep 1993;8(3):148-50. doi:10.1007/bf00341188

55. McCulloch PG, Blamey SL, Finlay IG, et al. A prospective comparison of gentamicin and metronidazole and moxalactam in the prevention of septic complications associated with elective operations of the colon and rectum. *Surgery, Gynecology & Obstetrics*. 1986;162(6):521-524.
56. McDermott FT, Polglase AL, Johnson WR, Hughes ESR. Prevention of wound infection in elective colorectal resections by preoperative cephazolin with and without metronidazole. *Australian and New Zealand Journal of Surgery*. 1981;51(4):351-353.
57. McLeish AR, Waxman B, Ross H, Allsop JR, Andrew JH, Bennett RC. Systemic Timentin(TM) is superior to oral tinidazole for antibiotic prophylaxis in elective colorectal surgery. *Diseases of the Colon and Rectum*. 1987;30(10):786-789.
58. Mehigan D, Zuidema GD, Cameron JL. The role of systemic antibiotics in operations upon the colon. *Surgery, Gynecology & Obstetrics*. 1981;153(4):573-576.
59. Mitchell NJ, Evans DS, Pollock D. Single dose metronidazole with and without cefuroxime in elective colorectal surgery. *Br J Surg*. Nov 1983;70(11):668-9. doi:10.1002/bjs.1800701109
60. Mittermayer H, Gross C, Brucke P. Single dose cefuroxime/metronidazole versus metronidazole alone in elective colorectal surgery. *American Surgeon*. 1984;50(8):418-423.
61. Morris DL, Fabricius PJ, Ambrose NS, Scammell B, Burdon DW, Keighley MR. A high incidence of bleeding is observed in a trial to determine whether addition of metronidazole is needed with latamoxef for prophylaxis in colorectal surgery. *Journal of Hospital Infection*. 1984;5(4):398-408. doi:10.1016/0195-6701(84)90008-2
62. Morris DL, Hares MM, Voogt RJ, Burdon DW, Keighley MR. Metronidazole need not be combined with an aminoglycoside when used for prophylaxis in elective colorectal surgery. *Journal of Hospital Infection*. 1983;4(1):65-69. doi:10.1016/0195-6701(83)90067-1
63. Morris DL, Wilson SR, Pain J, et al. A comparison of aztreonam/metronidazole and cefotaxime/metronidazole in elective colorectal surgery: antimicrobial prophylaxis must include gram-positive cover. *Journal of Antimicrobial Chemotherapy*. 1990;25(4):673-678. doi:10.1093/jac/25.4.673
64. Morris WT. Ceftriaxone is more effective than gentamicin/metronidazole prophylaxis in reducing wound and urinary tract infections after bowel operations. Results of a controlled, randomized, blind clinical trial. *Dis Colon Rectum*. Sep 1993;36(9):826-33. doi:10.1007/bf02047378
65. Morton AL, Taylor EW, Lindsay G, Wells GR. A multicenter study to compare cefotetan alone with cefotetan and metronidazole as prophylaxis against infection in elective colorectal operations. *Surg Gynecol Obstet*. Jul 1989;169(1):41-5.
66. Mosimann F, Cornu P, N'Ziya Z. Amoxycillin/clavulanic acid prophylaxis in elective colorectal surgery: a prospective randomized trial. *Journal of Hospital Infection*. 1997;37(1):55-64. doi:10.1016/s0195-6701(97)90073-6
67. Mozzillo N, Dionigi R, Ventriglia L. Multicenter study of aztreonam in the prophylaxis of colorectal, gynecologic and urologic surgery. *CHEMOTHERAPY*. 1989;35 Suppl 1:58-71.
68. Nyam D, Yeo M, Cheong D, Goh HS. Antibiotic prophylaxis in colorectal surgery: a randomised, double-blind, controlled trial of amoxycillin-clavulanic acid vs ceftriaxone and metronidazole. *Asian journal of surgery / Asian Surgical Association*. 1995;18(3):227-230.
69. Offer C, Weuta H, Bodner E. Efficacy of perioperative prophylaxis with ciprofloxacin or cefazolin in colorectal surgery. *Infection*. 1988;16 Suppl 1:S46-7. doi:10.1007/bf01650506
70. Ofstad E, Brabrand G, Helsingen N, et al. Tinidazole and doxycycline as antimicrobials in elective colorectal surgery. A randomized multicentre trial. *Scandinavian journal of gastroenterology Supplement*. 1980;59:29-35.
71. Olsen PR, Andersen HH, Hebjørn M, Pedersen VM, Hansen LK. The prophylaxis of metronidazole in colorectal surgery. *Dan Med Bull*. Sep 1983;30(5):345-8.
72. Pacelli F, Brisinda G, Bellantone R, Doglietto GB, Crucitti F. Single dose imipenem-cilastatin compared with three doses of cefuroxime and metronidazole as prophylaxis in elective colorectal surgery: a prospective randomized study. *J Chemother*. Dec 1991;3(6):372-5. doi:10.1080/1120009x.1991.11739123

73. Panichi G, Pantosti A, Giunchi G, et al. Cephalothin, cefoxitin, or metronidazole in elective colonic surgery? A single-blind randomized trial. *Diseases of the Colon and Rectum*. 1982;25(8):783-786. doi:10.1007/BF02553311
74. Perrott CA, Hinder RA, Cassel R, et al. Prophylactic antimicrobials in elective colorectal and biliary surgery. *South African Medical Journal Suid-Afrikaanse Tydskrif Vir Geneeskunde*. 1985;68(6):387-91.
75. Petrelli NJ, Conte CC, Herrera L, Stulc J, O'Neill P. A prospective, randomized trial of perioperative prophylactic cefamandole in elective colorectal surgery for malignancy. *Dis Colon Rectum*. Jun 1988;31(6):427-9. doi:10.1007/bf02552610
76. Plouffe JF, Perkins RL, Fass RJ, Carey LC, Macynski ME. Comparison of the effectiveness of moxalactam and cefazolin in the prevention of infection in patients undergoing abdominal operations. *Diagn Microbiol Infect Dis*. Jan 1985;3(1):25-31. doi:10.1016/0732-8893(85)90063-x
77. Reers B, Winde G, Sulkowski U, Blum M. Single-dose prophylaxis in elective colorectal surgery: a prospective, randomized trial with piperacillin or latamoxef. *Journal of chemotherapy (Florence, Italy)*. 1989;1(4 Suppl):997-998.
78. Renner H, Losch H. Experiences with ceftriaxone (rocephin) in perioperative antibiotic prophylaxis for elective colon surgery. *Journal of Chemotherapy*. 1989;1(4 Suppl):1005-6.
79. Rodolico G, Puleo S, Blandino G, et al. Aztreonam versus gentamicin for short-term prophylaxis in biliary and gastric surgery. *Reviews of Infectious Diseases*. 1991;13 Suppl 7:S616-20.
80. Roland M, Bergan T, Bjerkeset T, et al. Prophylactic regimens in colorectal surgery: comparisons between metronidazole used alone or with ampicillin for one or three days. *World J Surg*. Aug 1985;9(4):626-32. doi:10.1007/bf01656069
81. Rorbaek-Madsen M, Toftgaard C, Graversen HP, et al. Cefoxitin for one day vs. ampicillin and metronidazole for three days in elective colorectal surgery: A prospective, randomized, multicenter study. *Diseases of the Colon and Rectum*. 1988;31(10):774-777. doi:https://dx.doi.org/10.1007/BF02560105
82. Rowe-Jones DC, Peel AL, Kingston RD, Shaw JF, Teasdale C, Cole DS. Single dose cefotaxime plus metronidazole versus three dose cefuroxime plus metronidazole as prophylaxis against wound infection in colorectal surgery: multicentre prospective randomised study. *Bmj*. Jan 6 1990;300(6716):18-22. doi:10.1136/bmj.300.6716.18
83. Sato T, Takayama T, Fujii M, et al. Systemic use of antibiotics does not prevent postoperative infection in elective colorectal surgery: a randomized controlled trial. *Journal of Infection & Chemotherapy*. 2009;15(1):34-8. doi:https://dx.doi.org/10.1007/s10156-008-0660-x
84. Shatney CH. Antibiotic prophylaxis in elective gastro-intestinal tract surgery: a comparison of single-dose pre-operative cefotaxime and multiple-dose cefoxitin. *Journal of Antimicrobial Chemotherapy*. 1984;14 Suppl B:241-5.
85. Shimizu J, Ikeda K, Fukunaga M, et al. Multicenter prospective randomized phase II study of antimicrobial prophylaxis in low-risk patients undergoing colon surgery. *Surgery Today*. 2010;40(10):954-7. doi:https://dx.doi.org/10.1007/s00595-009-4176-5
86. Skipper D, Karran SJ. A randomized prospective study to compare cefotetan with cefuroxime plus metronidazole as prophylaxis in elective colorectal surgery. *Journal of Hospital Infection*. 1992;21(1):73-77. doi:https://dx.doi.org/10.1016/0195-6701%2892%2990155-F
87. Slama TG, Carey LC, Fass RJ. Comparative efficacy of prophylactic cephalothin and cefamandole for elective colon surgery: results of a prospective, randomized, double-blind study. *Am J Surg*. May 1979;137(5):593-6. doi:10.1016/0002-9610(79)90029-1
88. Solhaug JH, Bergman L, Kylberg F. A randomized evaluation of single dose chemoprophylaxis in elective colorectal surgery--a comparison between metronidazole and doxycycline. *Annals of Clinical Research*. 1983;15(1):15-20.
89. Stubbs RS, Griggs NJ, Kelleher JP, Dickinson IK, Moat N, Rimmer DM. Single dose mezlocillin versus three dose cefuroxime plus metronidazole for the prophylaxis of wound infection after large bowel surgery. *J Hosp Infect*. May 1987;9(3):285-90. doi:10.1016/0195-6701(87)90126-5

© 2026 Motaghi S et al. *JAMA Network Open*.

90. Taylor EW, Lindsay G, Bremner D, et al. Selective decontamination of the colon before elective colorectal surgery. *World Journal of Surgery*. 1994;18(6):926-932. doi:https://dx.doi.org/10.1007/BF00299111
91. TheNorwegianStudyGroup. Should antimicrobial prophylaxis in colorectal surgery include agents effective against both anaerobic and aerobic microorganisms? A double-blind, multicenter study. *Surgery*. 1985;97(4):402-408.
92. Thomas WE, Cooper MJ, Holt A, Reeves D. Latamoxef: single agent prophylaxis in colorectal surgery. *Journal of Antimicrobial Chemotherapy*. 1985;16(1):121-128. doi:10.1093/jac/16.1.121
93. Tornqvist A, Forsgren A, Leandoer L, Ursing J. Antibiotic treatment during surgery for diffuse peritonitis: a prospective randomized study comparing the effects of cefuroxime and of a cefuroxime and metronidazole combination. *British Journal of Surgery*. 1985;72(4):261-264. doi:https://dx.doi.org/10.1002/bjs.1800720404
94. Ulrich C. 24-hour systemic antibiotic prophylaxis in large-bowel surgery. *Netherlands journal of surgery*. 1981;33(1):3-9.
95. UoM. Clinical trial of prophylaxis of wound sepsis in elective colorectal surgery comparing ticarcillin with tinidazole. University of Melbourne Colorectal Group. *Aust N Z J Surg*. Mar 1986;56(3):209-13.
96. A comparison of single-dose systemic Timentin with mezlocillin for prophylaxis of wound infection in elective colorectal surgery. University of Melbourne Colorectal Group. Dis Colon Rectum. 1989 Nov;32(11):940-3. PMID: 2680356.
97. Utley RJ, Macbeth WAAG. Peroperative cefoxitin. A double-blind prospective study in the prevention of wound infections. *Journal of the Royal College of Surgeons of Edinburgh*. 1984;29(3):143-146.
98. Viddal KO, Semb LS. Tinidazole and doxycycline compared to doxycycline alone as prophylactic antimicrobial agents in elective colorectal surgery. *Scand J Gastroenterol Suppl*. 1980;59:21-4.
99. Walker AJ, Taylor EW, Lindsay G, Dewar EP. A multicentre study to compare piperacillin with the combination of netilmicin and metronidazole for prophylaxis in elective colorectal surgery undertaken in district general hospitals. *Journal of Hospital Infection*. 1988;11(4):340-348. doi:10.1016/0195-6701(88)90087-4
100. Watt-Boolsen S, Justesen T, Blichert-Toft M, Bech H. The prophylaxis of septic complications in colo-rectal surgery. A controlled trial of metronidazole and oxytetracycline. *Acta Chirurgica Scandinavica*. 1979;145(4):263-266.
101. Weidema WF, van den Boogaard AE, Wesdorp RI, van Boven CP, Greep JM. 24-hour systemic antimicrobial prophylaxis with gentamicin and metronidazole, or metronidazole alone, in elective colorectal surgery after mechanical bowel preparation with mannitol and whole gut irrigation. *Acta Chirurgica Belgica*. 1985;85(6):349-353.
102. Wetterfors J, Hoejer H. Prophylaxis with doxycycline (vibramycin) in colorectal surgery. *Scandinavian Journal of Gastroenterology*. 1980;15(Suppl 59):11-16.
103. Willis AT, Ferguson IR, Jones PH, et al. Metronidazole in prevention and treatment of bacteroides infections in elective colonic surgery. *British Medical Journal*. 1977;1(6061):607-610. doi:10.1136/bmj.1.6061.607
104. Wohlfahrt R, Siedek M. Perioperative infection prophylaxis in colon surgery. *Chemioterapia*. Jun 1987;6(2 Suppl):603-4.
105. Zanella E, Rulli F. A multicenter randomized trial of prophylaxis with intravenous cefepime + metronidazole or ceftriaxone + metronidazole in colorectal surgery. The 230 Study Group. *Journal of chemotherapy (Florence, Italy)*. 2000;12(1):63-71. doi:10.1179/joc.2000.12.1.63

© 2026 Motaghi S et al. *JAMA Network Open*.
