# Supplementary material for: Antibiotic Prophylaxis Strategies and Surgical Site Infections in Colorectal Surgery: A Systematic Review and Network Meta-Analysis
Source: JAMA Netw Open. 2026 Feb 19;9(2):e2560095. doi: 10.1001/jamanetworkopen.2025.60095 (PMC12921537; doi:10.1001/jamanetworkopen.2025.60095)
Supplement: Supplement 2. — Data Sharing Statement [file jamanetwopen-e2560095-s002.pdf]

Data Sharing Statement

Motaghi. Antibiotic Prophylaxis Strategies and Surgical Site Infections in Colorectal Surgery. *JAMA Netw Open*. Published February 19, 2026. doi:10.1001/jamanetworkopen.2025.60095

Data
Data available: No

Additional Information
Explanation for why data not available: We used aggregate data from publicly available published studies and are provided in the supplementary material.
